# cAMP activates calcium signalling via phospholipase C to regulate cellulase production in the filamentous fungus *Trichoderma reesei*

**DOI:** 10.1186/s13068-021-01914-0

**Published:** 2021-03-08

**Authors:** Yumeng Chen, Xingjia Fan, Xinqing Zhao, Yaling Shen, Xiangyang Xu, Liujing Wei, Wei Wang, Dongzhi Wei

**Affiliations:** 1 State Key Lab of Bioreactor Engineering, New World Institute of Biotechnology, East China University of Science and Technology, 130 Meilong Road , P.O.B. 311, Shanghai, 200237 China; 2 State Key Laboratory of Microbial Metabolism, Joint International Research Laboratory of Metabolic and Developmental Sciences, School of Life Sciences and Biotechnology, Shanghai Jiao Tong University, Shanghai, 200240 China; 3 Zaozhuang Jie Nuo Enzyme Co. Ltd., Shandong, China

**Keywords:** *Trichoderma reesei*, Cellulase, Adenylate cyclase, Calcium signalling, Cyclic AMP, Mn^2+^/DMF stimulation, Filamentous fungi

## Abstract

**Background:**

The filamentous fungus *Trichoderma reesei* is one of the best producers of cellulase and has been widely studied for the production of cellulosic ethanol and bio-based products. We previously reported that Mn^2+^ and *N*,*N*-dimethylformamide (DMF) can stimulate cellulase overexpression via Ca^2+^ bursts and calcium signalling in *T. reesei* under cellulase-inducing conditions. To further understand the regulatory networks involved in cellulase overexpression in *T. reesei*, we characterised the Mn^2+^/DMF-induced calcium signalling pathway involved in the stimulation of cellulase overexpression.

**Results:**

We found that Mn^2+^/DMF stimulation significantly increased the intracellular levels of cAMP in an adenylate cyclase (ACY1)-dependent manner. Deletion of *acy1* confirmed that cAMP is crucial for the Mn^2+^/DMF-stimulated cellulase overexpression in *T. reesei*. We further revealed that cAMP elevation induces a cytosolic Ca^2+^ burst, thereby initiating the Ca^2+^ signal transduction pathway in *T. reesei*, and that cAMP signalling causes the Ca^2+^ signalling pathway to regulate cellulase production in *T. reesei*. Furthermore, using a phospholipase C encoding gene *plc-e* deletion strain, we showed that the *plc-e* gene is vital for cellulase overexpression in response to stimulation by both Mn^2+^ and DMF, and that cAMP induces a Ca^2+^ burst through PLC-E.

**Conclusions:**

The findings of this study reveal the presence of a signal transduction pathway in which Mn^2+^/DMF stimulation produces cAMP. Increase in the levels of cAMP activates the calcium signalling pathway via phospholipase C to regulate cellulase overexpression under cellulase-inducing conditions. These findings provide insights into the molecular mechanism of the cAMP–PLC–calcium signalling pathway underlying cellulase expression in *T. reesei* and highlight the potential applications of signal transduction in the regulation of gene expression in fungi.

**Supplementary Information:**

The online version contains supplementary material available at 10.1186/s13068-021-01914-0.

## Background

Lignocellulosic biomass is one of the most abundant, renewable, and relatively inexpensive raw materials for biorefineries. This biomass is used for the production of value-added chemicals and fuels [1–3]. Filamentous fungi are the principal producers of hydrolytic enzymes dedicated to the degradation of lignocellulosic biomass [4–6]. The filamentous fungus *Trichoderma reesei* is one of the best-studied model organisms for the production of hydrolytic enzymes [7–9]. *T. reesei* harbours a variety of cellulase- and hemicellulase-encoding genes. Their expression is controlled by a complex regulatory network [8, 10]. A better understanding of the regulatory machinery of *T. reesei* at the molecular level will lead to new metabolic engineering approaches to construct strains capable of more efficient production of cellulase than is presently possible [5, 8].

Cellulase production is regulated by a complex signalling cascade and regulatory network [11]. The precise mechanism by which environmental signal-related stimulation regulates the expression of cellulases remains unclear, although key regulators in different signal transduction pathways have been identified recently [12–15]. Recent studies have demonstrated that stimulation of the calcium signal transduction pathway by environmental signals can affect cellulase production and cell metabolism in fungi [12, 16–20]. Chen et al. [12] demonstrated that external Ca^2+^ stimulated hyphal growth, growth-independent cellulase production, and total protein secretion of *T. reesei* Rut-C30 involving the Ca^2+^/calmodulin–calcineurin–CRZ1 signal transduction pathway. Other authors have reported that high levels of Ca^2+^ and Mn^2+^ concentrations generate a prolonged nuclear accumulation of CrzA in *Aspergillus nidulans* [16, 17]. Martins-Santana et al. [18] demonstrated that Ca^2+^ acts synergistically with CRZ1 to modulate cellulase gene expression in *T. reesei*. We previously reported that Mn^2+^ and the organic solvent *N*,*N*-dimethylformamide (DMF) can stimulate cellulase overexpression via a calcium signal transduction pathway in *T. reesei* [19, 20]. Above all, cellular Ca^2+^, which is a ubiquitous second messenger, is an important intracellular signalling molecule involved in the regulation of gene expression in fungi stimulated by environmental signals. However, the details of the signal transduction pathway from environmental stimulation to calcium signalling in regulating cellulase gene expression remain unknown in filamentous fungi.

Extensive progress has been made in understanding the importance of the other ubiquitous second messenger, cyclic AMP (cAMP), in filamentous fungi. Exogenous cAMP leads to increased endoglucanase synthesis in *T. reesei* [21]. A positive correlation was observed between intracellular cAMP concentration and cellulase expression levels [21, 22]. Adenylate cyclase is a crucial component of the cAMP pathway that generates cAMP. *T. reesei* adenylate cyclase (ACY1) has a consistently positive effect on cellulase gene expression [14]. As a secondary messenger, cAMP is also involved in responses to extracellular signals, such as blue light [23] and carbon metabolism [24]. However, the cAMP signal transduction pathway that regulates cellulase gene expression under environmental stimulation in filamentous fungi has not yet been fully elucidated.

In this study, we assessed the signal transduction pathway from Mn^2+^/DMF stimulation to calcium signalling and cellulase production in *T. reesei* under cellulase-inducing conditions. Our results demonstrate that phospholipase C is an important link between cAMP and calcium signalling in cellulase production that occurs in response to Mn^2+^/DMF stimulation. These findings provide evidence for the mechanism of the environmental regulation of cellulase expression in filamentous fungi.

## Results

### Mn^2+^/DMF stimulation produces cAMP in an ACY1-dependent manner

We previously demonstrated that a biologically relevant level of extracellular Mn^2+^ or DMF markedly stimulates cellulase overexpression in *T. reesei* [19, 20]. As a secondary messenger, cAMP is involved in responses to extracellular signals. To examine whether Mn^2+^/DMF stimulation had any effect on cAMP concentration in *T. reesei*, precultured mycelia of *T. reesei* QM6a was inoculated into fresh liquid MM containing 1% Avicel (cellulase-inducing conditions) as the sole carbon source with no further supplementation, or with the addition of 10 mM Mn^2+^ or 1% DMF, as previously described [19].

**Fig. 1:**
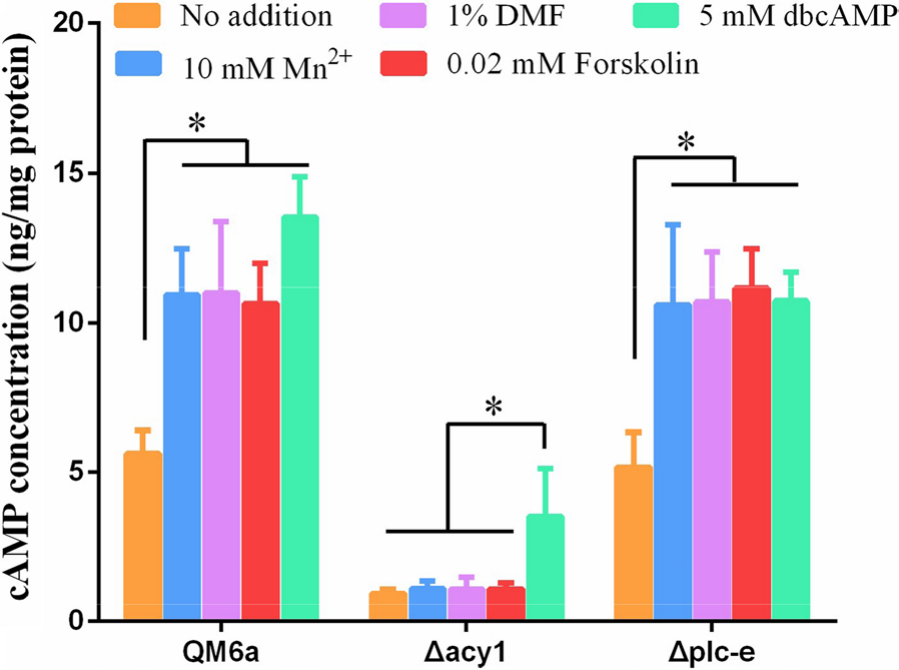
Intracellular cAMP concentration in *T. reesei* QM6a, Δ*acy1* and Δ*plc-e* strains under different conditions. The QM6a, Δ*acy1*, and Δ*plc-e* strains were cultured in MM with 2% glucose as a carbon source and then inoculated into fresh MM supplemented with 10 mM Mn^2+^, 1% DMF, 0.02 mΜ Forskolin, or 5 mM dbcAMP with 1% Avicel as the carbon source. Cultures with no added compounds were used as controls. Intracellular cAMP concentration was detected as described in the Materials and Methods. Values are the mean ± SD of the results from three independent experiments. Asterisks indicate significant differences from the control (*p < 0.05, Student’s *t-*test)

The intracellular cAMP concentrations of *T. reesei* QM6a after the addition of Mn^2+^ and DMF are presented in Fig. 1. Stimulation with 10 mM Mn^2+^ produced a 76.5% increase in the intracellular cAMP concentration compared with that of the control (no addition) under the same growth conditions. Similarly, stimulation with 1% DMF resulted in a 74% increase in intracellular cAMP concentration compared with the control. These results showed that Mn^2+^/DMF stimulation could induce cAMP accumulation in *T. reesei*. Supplementation with forskolin, a direct adenylate cyclase activator [25], also increased the formation of intracellular cAMP by 50% compared with that of the control (Fig. 1). These results indicate that Mn^2+^/DMF stimulation can result in cAMP accumulation.

To further clarify whether the increased cAMP was mediated by the adenylate cyclase under Mn^2+^/DMF stimulation, we constructed an adenylate cyclase gene *acy1* deletion strain (Δ*acy1*) to detect its function during cAMP accumulation following Mn^2+^/DMF stimulation. The growth rate of the Δ*acy1* strains was clearly slower than that of the wild-type strain QM6a (Additional File 1: Figure S1), which is consistent with data from Schuster et al. [14]. We then compared the intracellular cAMP concentration in the Δ*acy1* strain under different conditions. Mn^2+^/DMF or Forskolin supplementation did not induce cAMP accumulation in the Δ*acy1* strains, which differed from the wild-type strain QM6a (Fig. 1). However, exogenous application of dbcAMP increased the cAMP concentration in both Δ*acy1* and wild-type QM6a (Fig. 1). The results indicated that Mn^2+^/DMF stimulation can result in cAMP accumulation in an ACY1-dependent manner.

### cAMP signalling mediates Mn^2+^/DMF-stimulated cellulase overexpression in ***T. reesei***

We found that the concentration of cytosolic cAMP was increased by Mn^2+^/DMF stimulation (Fig. 1). We thus examined whether cAMP signalling was responsible for Mn^2+^/DMF-stimulated cellulase overexpression.

**Fig. 2:**
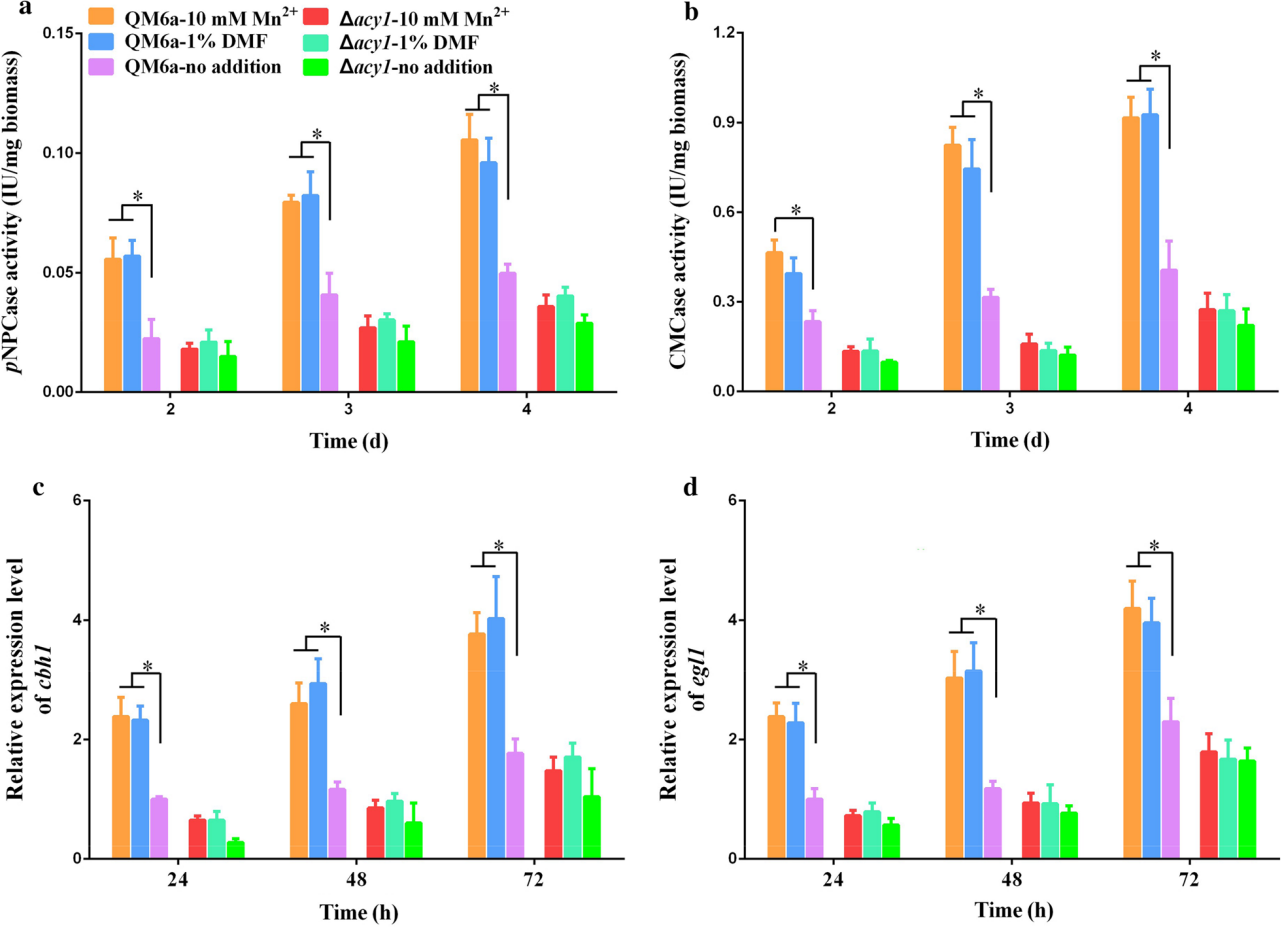
Effect of ACY1 on Mn^2+^/DMF-stimulated cellulase overexpression**. a** and** b**. *p*NPCase activity/mg biomass (**a**) and CMCase activity/mg biomass (**b**) of *T. reesei* QM6a and Δ*acy1* strains supplemented with 10 mM Mn^2+^ or 1% DMF. **c** and **d**. The relative expression levels of *cbh1* (**c**) and *egl1* (**d**) in *T. reesei* QM6a and Δ*acy1* strains cultured in medium supplemented with 10 mM Mn^2+^ or 1% DMF. No addition (QM6a-no addition and Δ*acy1*-no addition) were used as controls. The QM6a culture with no addition at 24 h was used as the reference sample. Values are the mean ± SD of the results from three independent experiments. Asterisks indicate significant differences from the control (*p < 0.05, Student’s *t* test)

To explore the role of cAMP signalling in Mn^2+^/DMF stimulation, cellulase production in the Δ*acy1* strains in response to Mn^2+^ and DMF supplementation was detected. As shown in Fig. 2a and b, supplementation with 10 mM Mn^2+^ or 1% DMF led to an almost 2.5-fold improvement in cellulase production (CMCase and *p*NPCase activities) in wild-type QM6a, but did not result in increased cellulase production in the Δ*acy1* strains. The *p*NPCase activity in the *acy1* re-complementation strain R*acy1* was similar to that in QM6a (Additional File 2: Figure S2). There was no obvious difference in CMCase or *p*NPCase activities with and without Mn^2+^/DMF addition in the Δ*acy1* strains (Fig. 2a, b).

RT-qPCR was performed to determine the transcription levels of the main cellulase genes *cbh1* and *egl1* in the QM6a and Δ*acy1* strains in response to Mn^2+^/DMF addition. In agreement with the levels of CMCase and *p*NPCase activities, deletion of *acy1* abolished the Mn^2+^/DMF-stimulated overexpression of cellulase genes compared with the wild-type strain QM6a at all time points (Fig. 2c, d). Cellulase production was detected in the Δ*acy1* strains in response to exogenous dbcAMP or dbcAMP in combination with Mn^2+^ and DMF. As shown in Additional File 3: Figure S3, supplementation with 5 mM dbcAMP led to a significant improvement in cellulase production (CMCase and *p*NPCase activities) in both Δ*acy1* strains and wild-type QM6a.

These results indicate that cAMP signalling mediates Mn^2+^/DMF-stimulated cellulase overexpression in *T. reesei*.

### cAMP elevation can induce cytosolic Ca^2+^ burst and Ca^2+^ signalling

Our previous results suggested that calcium signalling is the key reason for the Mn^2+^/DMF-stimulated cellulase overexpression in *T. reesei* [19, 20]. Presently, cAMP signalling also appeared to mediate Mn^2+^/DMF-stimulated cellulase overexpression (Fig. 2). To gain insight into the relationship between cAMP and calcium signalling, the effect of cAMP on the cytosolic Ca^2+^ was examined by the use of Fluo-4 AM, a Ca^2+^-specific fluorescent probe [26].

**Fig. 3:**
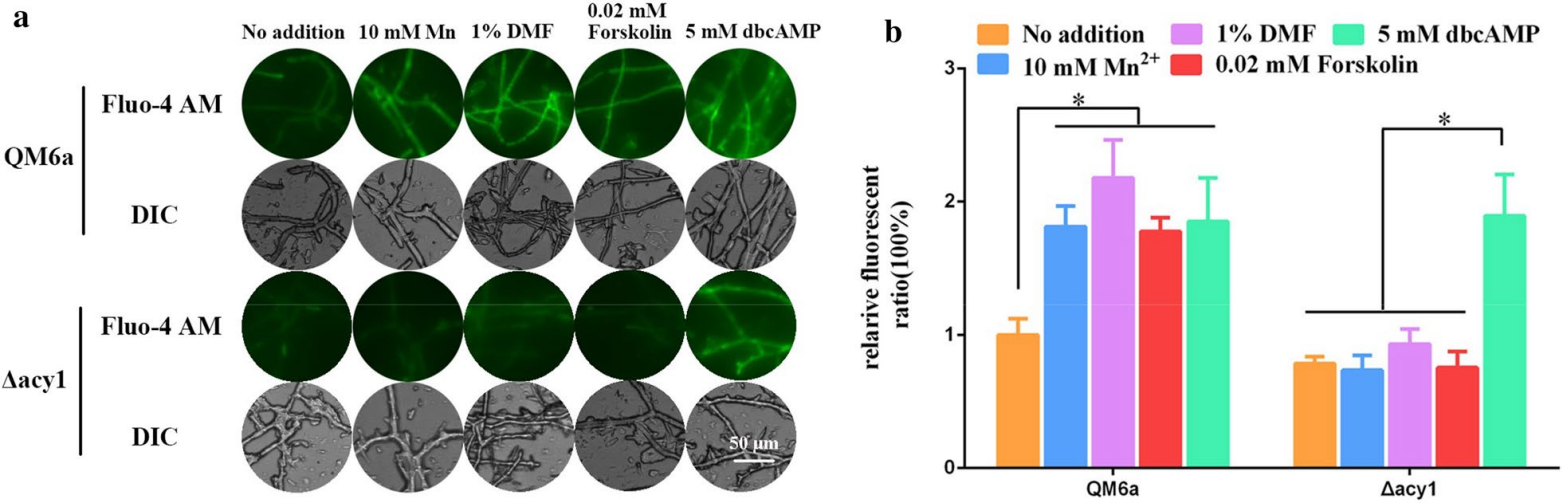
Cytosolic Ca^2+^ levels in *T. reesei* QM6a and Δ*acy1* strains under different conditions.** a** The analysis of cytosolic Ca^2+^ levels using the Ca^2+^ fluorescent probe Fluo-4 AM. The QM6a and Δ*acy1* strains were cultured in MM with 2% glucose as the carbon source, then inoculated to fresh MM supplemented with 10 mM Mn^2+^, 1% DMF, 0.02 mΜ Forskolin, or 5 mM dbcAMP with 1% Avicel as the carbon source. Cultures with no addition was used as the controls. Fluo-4 AM (50 μM) was used for detection. The intensity was monitored using automatic inverted fluorescence microscopy. Green fluorescence intensity represents the free cytosolic Ca^2+^ levels. DIC, differential interference contrast. The control images of QM6a were also used in Fig. 5a. **b** Comparative fluorescence ratio analysis of different supplementation on cytosolic Ca^2+^ levels in *T. reesei* QM6a and Δ*acy1* strains. The y-axis represents the Ca^2+^ fluorescence ratio measured by CLSM, and the x-axis represents the different strains tested. Values are the means ± SEM of the results from three independent experiments. Asterisks indicate significant differences from the control (*p < 0.05, Student’s *t* test)

As illustrated in Fig. 3a and b, the addition of Mn^2+^, DMF, Forskolin, and dbcAMP induced a Ca^2+^ burst in wild-type QM6a by increasing cytosolic Ca^2+^ to concentrations from 70 to 100% higher than that in cells not cultured with Mn^2+^, DMF, Forskolin, or dbcAMP. As noted above, Mn^2+^, DMF, forskolin, and dbcAMP significantly increased the concentration of cytosolic cAMP in QM6a (Fig. 1). These data suggested that a high concentration of cytosolic cAMP is associated with increased cytosolic Ca^2+^ content (Figs. 1 and 3).

The effect of cAMP on cytosolic Ca^2+^ was also examined in Δ*acy1* strains. As shown in Fig. 3a and b, Mn^2+^, DMF, and forskolin did not increase the cytosolic Ca^2+^ concentration in the Δ*acy1* strains. However, dbcAMP resulted in an approximately 80% increase in cytosolic Ca^2+^ concentration in Δ*acy1* strains compared with the concentration in the absence of any added compound (Fig. 3a, b). Thus, when cAMP synthesis is blocked in Δ*acy1* strains, Mn^2+^, DMF, and forskolin addition cannot induce cytosolic Ca^2+^ burst, while exogenous dbcAMP addition can. This is because dbcAMP can still increase the cytosolic cAMP concentration in Δ*acy1* strains, while Mn^2+^, DMF, and Forskolin cannot (Fig. 1). These data suggested that Mn^2+^/DMF stimulation can increase cytosolic cAMP content, which induces cytosolic Ca^2+^ burst in *T. reesei* QM6a.

The effect of cAMP on Ca^2+^ signalling was further examined in the QM6a and Δ*acy1* strains. As shown in Additional File 4: Figure S4, the addition of Mn^2+^, DMF, and forskolin significantly upregulated the expression levels of three Ca^2+^ signalling genes (*cam*, *cna*, and *crz1*) in wild-type QM6a compared to untreated cells. However, the expression levels of *cam*, *cna1*, and *crz1* remained stable in *acy1* deletion strains, irrespective of whether Mn^2+^, DMF, and forskolin were added to the cells. The addition of dbcAMP resulted in an increase in the expression levels of *cam*, *cna1*, and *crz1* in both wild-type QM6a and Δ*acy1* strains compared to untreated cells. These data suggest that an increase in cAMP levels induces a cytosolic Ca^2+^ burst that activates the Ca^2+^ signal transduction pathway in *T. reesei*.

To further investigate the link between cAMP and Ca^2+^ signalling, we measured cellulase production in response to exogenous dbcAMP and dbcAMP in combination with LaCl_3._ LaCl_3_ is a plasma membrane Ca^2+^ channel blocker that inhibits the cytoplasmic Ca^2+^ burst. As shown in Additional File 5: Figure S5, supplementation with 5 mM dbcAMP led to a significant increase in cellulase production (CMCase and *p*NPCase activities) in wild-type QM6a. However, cellulase activities decreased significantly when LaCl_3_ supplements were added compared to the no-LaCl_3_ control. These results suggest that cAMP signalling causes Ca^2+^ signalling to regulate cellulase production in *T. reesei*.

### PLC-E mediates both Mn^2+^- and DMF-stimulated cellulase overexpression

To gain further insight into the mechanism by which Mn^2+^/DMF stimulates cellulase overexpression, we compared the transcriptomes of three *T. reesei* QM6a cultures, with no addition, with the addition of 10 mM Mn^2+^, or with the addition of 1% DMF (liquid MM containing 1% Avicel as the sole carbon source) incubated at 28 °C and 200 rpm for 36 h [20]. This resulted in the retrieval of 846 genes that were differentially expressed following 10 mM Mn^2+^ addition compared to the control (no addition). Of these 846 genes, 580 were up-regulated and 266 were down-regulated (Additional File 6: Figure S6, Additional File 7: Table S1). In our previous study, we compared the transcriptomes of *T. reesei* QM6a cultured with no additions and the addition of 1% DMF. We observed that 81 genes were upregulated and 21 were downregulated following 1% DMF addition, compared to the control [20]. In the current study, we analysed the overlap between the differentially expressed genes regulated by Mn^2+^ and those in DMF. Sixty-three genes were differentially expressed in the presence of both Mn^2+^ and DMF (Additional File 8: Table S2). Of these genes, 56 were upregulated and 7 genes were downregulated by both Mn^2+^ and DMF compared with no addition. The same genes were up- or down-regulated under Mn^2+^/DMF stimulation, implying a similar putative mechanism of cellular overexpression by Mn^2+^/DMF.

Of the genes that were consistently differentially expressed by both Mn^2+^ and DMF, 11 cellulose degradation-related genes were significantly upregulated in the presence of 1% DMF or 10 mM Mn^2+^ (Additional File 9: Table S3). Two main cellobiohydrolase-encoding genes (*cbh1* and *cbh2*; IDs: 123,989 and 72,567), three endoglucanase-encoding genes (IDs: 122,081, 120,312, and 49,976), including two main endoglucanase genes (*egl1* and *egl2*), one beta-glucosidase-encoding gene (*cel3b*; ID: 121,735), a xylanase-encoding gene (*xyn3*; ID: 120,229), and transcription of accessory protein-encoding genes, including those of swollenin gene *swo1* (ID: 123,992) and *cip2* (ID: 123,940) [7], were consistently upregulated in response to 10 mM Mn^2+^ and DMF addition. The transcriptome data agreed with our previous cellulase activity and qPCR results [19, 20].

**Fig. 4:**
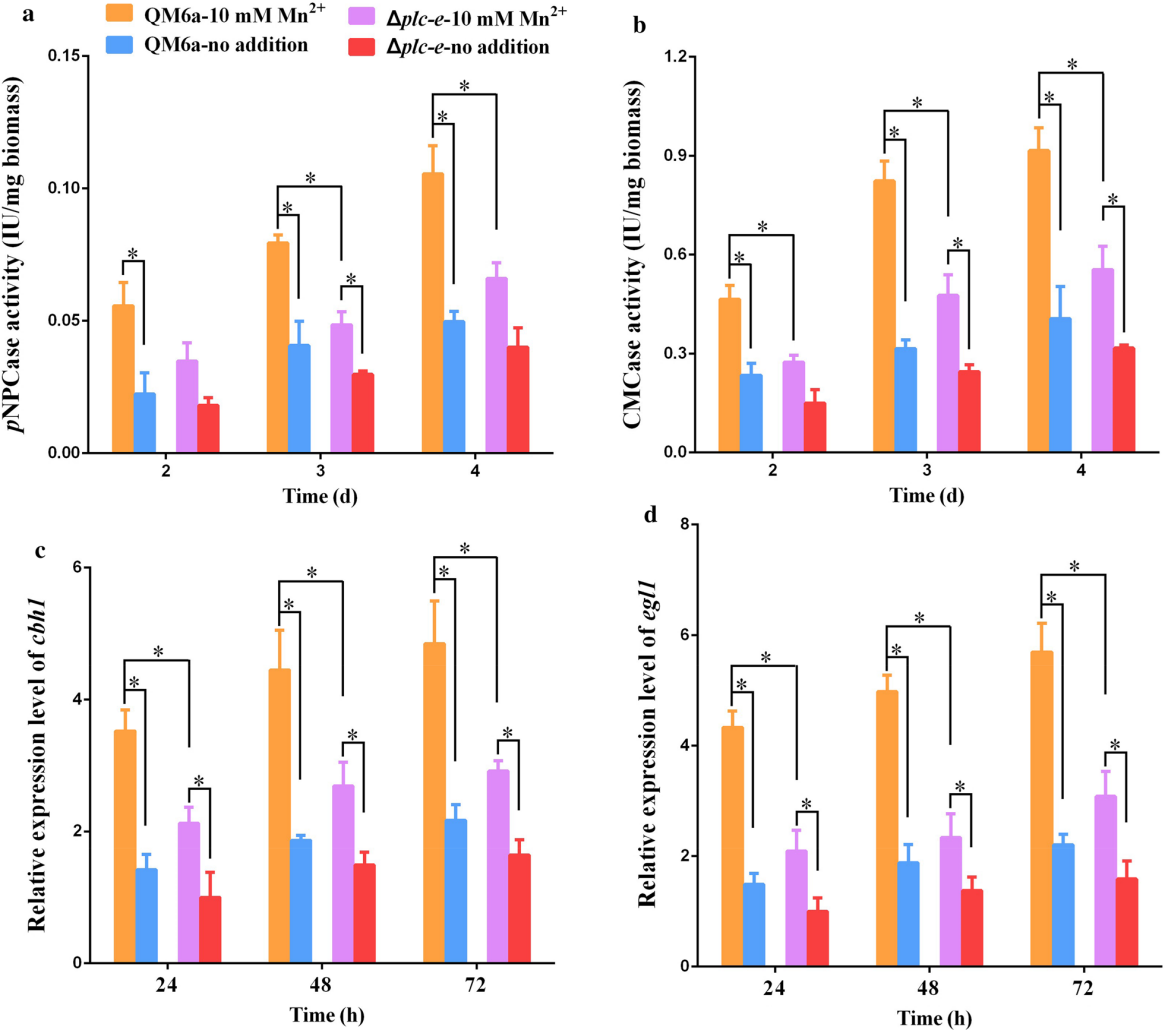
Effect of PLC-E on Mn^2+^-stimulated cellulase overexpression.** a** and **b**. *p*NPCase activity/mg biomass (**a**) and CMCase activity/mg biomass (**b**) of *T. reesei* QM6a and Δ*plc-e* strains supplemented with 10 mM Mn^2+^. **c** and **d** The relative expression levels of *cbh1* (**c**) and *egl1* (**d**) in *T. reesei* QM6a and Δ*plc-e* strains cultured in medium supplemented with 10 mM Mn^2+^. No addition (QM6a-no addition and Δ*plc-e*-no addition) was used as the respective control. The Δ*plc-e*-no addition at 24 h was used as the reference sample. Values are the mean ± SD of the results from three independent experiments. Asterisks indicate significant differences from the control (*p < 0.05, Student’s *t* test)

Of the genes that were consistently differentially expressed by both Mn^2+^ and DMF, the transcriptional levels of *plc-e*, which encodes a phospholipase C protein, were significantly upregulated in response to 10 mM Mn^2+^ and DMF addition (Additional File 10: Table S4 and [20]). Phospholipase C activity is related to calcium release from intracellular pools, which increases the concentration of cytosolic Ca^2+^ resulting in a cytosolic Ca^2+^ burst [11]. We previously reported that the transcriptional level of *plc-e* was remarkably upregulated in DMF-stimulated strains and suggested that PLC-E is involved in DMF-stimulated cellulase overexpression [20]. The latest results implied that PLC-E might also participate in Mn^2+^-stimulated cellulase overexpression. Thus, we further examined the role of PLC-E in cellulase overexpression under Mn^2+^ addition. As shown in Fig. 4, the effect of Mn^2+^ stimulation on cellulase production was remarkably reduced in the Δ*plc-e* mutant, which was constructed in our previous study [20]. A marked increase in cellulase production (Fig. 4a, b) and the transcription levels of the main cellulase genes (Fig. 4c, d) stimulated by Mn^2+^ were observed in the wild-type QM6a, while the effect of Mn^2+^ stimulation on cellulase expression was remarkably reduced in the *plc-e* deletion strain at all time points (Fig. 4). The growth rate related to cellulase activity and transcription levels of *cbh1* and *egl1* after Mn^2+^ stimulation in the Δ*plc-e* mutant were significantly decreased compared with those in wild-type QM6a (Additional File 11: Figure S7). The *p*NPCase activity in the *plc-e* re-complementation strain R*plc-e* was similar to that in QM6a (Additional File 2: Figure S2). These results indicate that PLC-E is vital for cellulase overexpression in response to both Mn^2+^ and DMF stimulation.

### PLC-E exerts an important link between cAMP and Ca^2+^ signalling in the expression of cellulase

Earlier studies revealed that extracellular signals can activate phospholipase C (PLC), which is correlated with calcium signalling [11, 27]. Presently, the Ca^2+^ burst depended on the accumulation of cAMP, and PLC-E was a mediator in Mn^2+^/DMF-stimulated cellulase overexpression (Figs. 3, 4) and [20]. Additionally, the cytosolic cAMP concentration in the Δ*plc-e* strains showed no obvious change compared with that of QM6a under Mn^2+^/DMF stimulation, forskolin supplementation, or exogenous dbcAMP addition (Fig. 1). Thus, we hypothesised that PLC-E is an important link between cAMP and Ca^2+^.

**Fig. 5:**
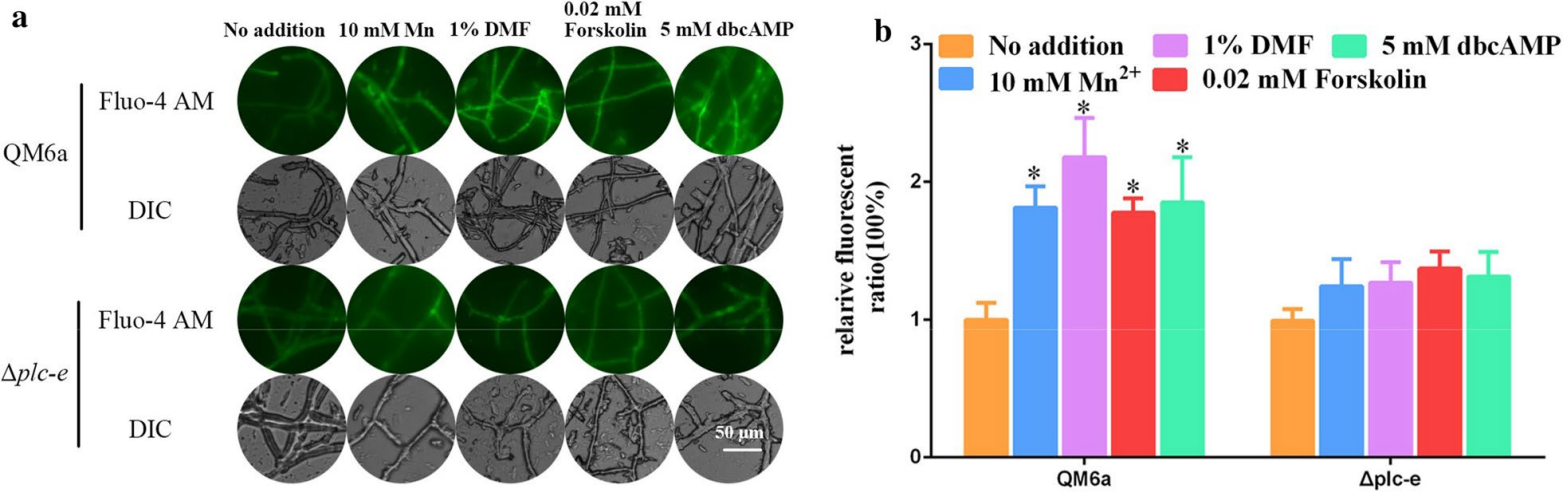
Cytosolic Ca^2+^ levels in *T. reesei* QM6a and Δ*plc-e* strains under different addition conditions. **a** The analysis of cytosolic Ca^2+^ levels using the Ca^2+^ fluorescent probe Fluo-4 AM. The QM6a and Δ*plc-e* strains were cultured in MM with 2% glucose as the carbon source, then inoculated into fresh MM supplemented with 10 mM Mn^2+^, 1% DMF, 0.02 mΜ Forskolin, or 5 mM dbcAMP with 1% Avicel as the carbon source. No addition was used as the controls. For detection, 50 μM Fluo-4 AM was used. Intensity was monitored using automatic inverted fluorescence microscopy. Green fluorescence intensity represents the free cytosolic Ca^2+^ levels. DIC, differential interference contrast. The control images of QM6a were also used in Fig. 3a. **b** Comparative fluorescence ratio analysis of different addition on cytosolic Ca^2+^ levels in *T. reesei* QM6a and Δ*plc-e* strains. The y-axis represents the Ca^2+^ fluorescence ratio measured by CLSM, and the x-axis represents the different strains tested. Values are the means ± SEM of the results from three independent experiments. Asterisks indicate significant differences from the control (*p < 0.05, Student’s *t* test)

To clarify whether PLC-E is involved in Ca^2+^ bursts induced by cAMP, the Ca^2+^ concentration was compared between the QM6a and Δ*plc-e* strains treated with Mn^2+^, DMF, forskolin, and dbcAMP. The cytosolic Ca^2+^ levels remained almost stable or were only slightly enhanced in the Δ*plc-e* strains in the presence of Mn^2+^/DMF stimulation, forskolin supplementation, or exogenous dbcAMP addition. A significant Ca^2+^ burst was observed in wild-type QM6a under all these conditions compared with that of the untreated control (Fig. 5a, b). The cytosolic Ca^2+^ burst induced by cAMP was significantly weakened in the Δ*plc-e* strains. These results suggest that PLC-E mediates the Ca^2+^ burst induced by cAMP. Additionally, the cytosolic cAMP concentration in the Δ*plc-e* strains was similar to that of QM6a under conditions of Mn^2+^/DMF stimulation, forskolin supplementation, or exogenous dbcAMP addition (Fig. 1). These results suggest that cAMP induces Ca^2+^ bursts through PLC-E. Furthermore, the significantly upregulated expression levels of three Ca^2+^ signalling genes (*cam*, *cna* and *crz1*) in wild-type QM6a induced by cAMP were significantly weakened in the Δ*plc-e* strains (Additional File 12: Figure S8). The collective results implicated PLC-E as an important link between cAMP and Ca^2+^ signalling in cellulase expression.

## Discussion

The cAMP pathway is a central signalling cascade with crucial functions in all organisms [11]. We found that in response to Mn^2+^/DMF stimulation, the intracellular cAMP concentration was significantly increased and that the cAMP elevation was responsible for Mn^2+^/DMF-stimulated cellulase overexpression (Figs. 1 and 2). These results were similar to the reports that the formation of cellulase can be altered by the addition of cAMP in *T. reesei* [21, 28]. We further obtained evidence that adenylate cyclase ACY1, the central component of the cAMP pathway, mediates Mn^2+^/DMF-stimulated cAMP accumulation and cellulase overexpression (Figs. 1 and 2). These data provide insights into the role of cAMP signalling in response to Mn^2+^/DMF stimulation.

Communication between cells and the environment is crucial for the survival of organisms. Cell surface receptors, such as G-protein-coupled receptors (GPCRs), act as sensors to connect to the environment [11, 29]. GPCRs react to a variety of extracellular cues and influence numerous regulatory pathways via the heterotrimeric G-protein signalling cascade, which plays a central role in signal transduction in filamentous fungi [11]. Some G proteins can activate adenylyl cyclase, resulting in increased cAMP production [30]. The cAMP signal is a well-known downstream target of Gα subunits in filamentous fungi [31] and can cross-talk with other signalling pathways, including calcium signalling. In *Saccharomyces cerevisiae*, GPCR Gpr1 interacts with Gpa2 and is required for stimulation of cAMP synthesis [32]. In *Cryptococcus neoformans*, GPCR Gpr4 interacts with Gα protein Gpa1 to regulate downstream elements of the cAMP pathway [33]. In *T. reesei*, Gα protein GNA3 is involved in the control of cAMP concentrations [22]. These data indicate that the cAMP pathway represents the main output of GPCRs and G-protein signalling. A transcriptome study found that seven GPCR-encoding genes were upregulated in the presence of 10 mM Mn^2+^. The seven genes included one GPCR *Tr72004* affiliated with the cAMP receptor-like family, four PTH11-type GPCR-encoding genes (*Tr110339*, *Tr124113*, *Tr121990*, *Tr62462*), and two GprK-type GPCR-encoding genes (*Tr37525* and *Tr81383*) (Additional File 11: Table S5). Presently, one GprK-type GPCR-encoding gene (*Tr81383*) was significantly upregulated in the presence of 1% DMF (Additional File 13: Table S5). These GPCRs might relate to adenylate cyclase ACY1 activation and intracellular cAMP accumulation in the presence of Mn^2+^ and DMF (Fig. 1). These data implied the complex transduction of signals in *T. reesei* via the GPCR cascade under Mn^2+^/DMF stimulation.

**Fig. 6:**
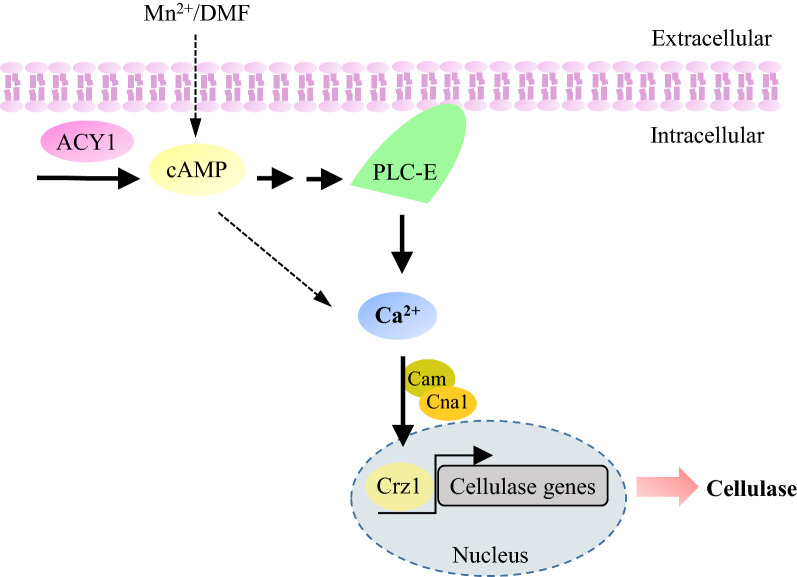
Mechanistic model of the cAMP and calcium signalling pathway in cellulase overexpression responding to Mn^2+^/DMF stimulation in *T. reesei* under cellulase-inducing conditions. Mn^2+^/DMF stimulation induces intracellular cAMP accumulation in an adenylate cyclase (ACY1)-dependent manner. An increase in cAMP levels induces a cytosolic Ca^2+^ burst that stimulates the Ca^2+^ signal transduction pathway to regulate cellulase expression in *T. reesei* under cellulase-inducing conditions. PLC is vital for cellulase overexpression in response to stimulation by both Mn^2+^ and DMF. cAMP induces Ca^2+^ bursts via PLC-E. The solid arrows indicate data supported by our own experiments, and the dashed arrows indicate other unknown regulation pathways

Calcium and calcium signalling play crucial roles in intracellular signalling processes in lower eukaryotes [11, 27]. Calcium signalling is mediated by the cytosolic Ca^2+^ concentration to activate appropriate downstream responses [27, 34]. The calcium signalling pathway interacts with other signalling pathways, such as cAMP signalling, alkaline pH signalling, and reactive oxygen species [27, 35–37]. In the present study, cross-talk was evident between cAMP and Ca^2+^ signals under Mn^2+^/DMF^−^stimulated cellulase overexpression (Fig. 6). Similar results were reported in a previous study, in which an increase in cAMP mobilised Ca^2+^ from intracellular stores and also activated the influx of Ca^2+^ from the extracellular medium in *Plasmodium falciparum* [35]. Another similar study showed that cAMP-mediated phosphorylation regulated calcium homeostasis by activating calcium channels in *Aspergillus niger* [38]. The collective prior and present results indicate a highly complex relationship between the Ca^2+^ and cAMP signalling pathways in the regulation of the expression of the corresponding genes under different environmental signals. This study demonstrates that PLC is an important link between cAMP and calcium signalling pathways in cellulase expression. However, other cross-talk between these two pathways remains unclear and requires further investigation.

Early studies revealed that PLC is related to calcium signalling [11, 27]. Extracellular signals can activate PLC and increase inositol-1,4,5-trisphosphate (IP3) levels, leading to the release of Ca^2+^ from internal stores, resulting in a cytosolic Ca^2+^ burst [11, 39]. G proteins and cAMP can activate PLC, which in turn induces Ca^2+^ release [40, 41]. In *B. cinerea*, activation of calcium signalling by increased cytosolic Ca^2+^ mediated by G proteins and PLC has been observed [41]. Early studies indicated that cAMP activates Epac1, which in turn activates PLC and ensures calcium signalling activation [40]. Cellular calcium homeostasis is regulated by cAMP-mediated protein kinase A-dependent phosphorylation. The present data suggest that PLC-E is responsible for the increase in cytosolic Ca^2+^ and cellulase overexpression induced by cAMP in response to Mn^2+^/DMF stimulation in *T. reesei* (Fig. 6). The mechanism of cAMP activates PLC-E requires further investigation. Deletion of *plc-e* reduced the resulting cellulase activity upon the addition of Mn^2+^/DMF, not completely blocked the resulting cellulase activity upon the addition of Mn^2+^/DMF (Fig. 4), and the cytosolic Ca^2+^ levels were slightly elevated in the Δ*plc-e* strains when exogenous dbcAMP was added (Fig. 5). These results implied the presence of a second signal transduction pathway from cAMP to Ca^2+^, except *plc-e* (Fig. 6). These findings provide insights into the mechanism of the cAMP-PLC-calcium signalling pathway in response to environmental stimulation. The findings also indicate that the regulatory mechanisms of cellulase expression involve a complex signalling network in fungi.

## Conclusions

In summary, cellulase production is regulated by complex signal transduction pathways in response to environmental signal stimulation. We studied the signal transduction pathway, in which Mn^2+^/DMF stimulation increased cAMP levels in an ACY1-dependent manner. cAMP then induces a Ca^2+^ burst through PLC-E to improve cellulase expression in *T. reesei* (Fig. 6). These findings shed new light on the molecular mechanism of the cAMP–PLC–calcium signalling pathway underlying cellulase expression in filamentous fungi.

## Methods

### Strains and growth conditions

*Escherichia coli* DH5α was used for plasmid amplification. *Agrobacterium tumefaciens* strain AGL-1 was used for fungal transformation [42]. *T. reesei* QM6a (ATCC 13631) was used throughout the study. *E. coli* and *A. tumefaciens* were cultured in Luria broth (LB) medium. All strains of *T. reesei* were maintained on potato dextrose agar (PDA) plates at 28 °C. Conidia were collected from the PDA plates. All strains were cultured in the dark.

Minimal medium (MM; urea 0.3 g/L; (NH_4_)_2_SO_4_ 5 g/L; KH_2_PO_4_ 15 g/L; MgSO_4_ 0.6 g/L; CaCl_2_ 0.6 g/L; FeSO_4_**·**7H_2_O 5 mg/L; ZnSO_4_**·**7H_2_O 1.4 mg/L; CoCl_2_**·**6H_2_O 2 mg/L; pH 5.5) with 2% glucose or 1% Avicel was used to assess hyphal growth and cellulase production. Conidia (2 × 10^6^) were cultivated at 28 °C (200 rpm) in 50 mL of MM (2% glucose as the sole carbon source) for 36–48 h. The mycelia were inoculated into 100 mL of freshly prepared MM containing 1% Avicel as the sole carbon source with no further addition or the addition of Mn^2+^ (final concentration 10 mM), DMF (final concentration 1%), forskolin (final concentration 10 µM; Beyotime, Shanghai, China), or dibutyryl cAMP (dbcAMP, final concentration 5 mM; Sigma-Aldrich, St. Louis, MO, USA) as described previously [19]. Mycelia were grown for 96 h at 28 °C. One millilitre of culture liquid was collected at 24-h intervals to perform the assays.

### Enzymatic activity

Cellulase activity was measured as previously described [19]. In brief, the *p*NPCase activity was determined against 5 mM *p*-nitrophenol-D-cellobioside (*p*NPC, Sigma-Aldrich) as the substrate in 50 mM sodium acetate buffer at pH 5.0 and 50 °C for 30 min. One unit of *p*NPCase activity was defined as 1 μmol of *p*-nitrophenol released per min. CMCase activity was determined using 1% carboxymethylcellulose (CMC, Sigma-Aldrich) as the substrate in 50 mM sodium acetate buffer at pH 5.0, and 50 °C for 30 min. One unit of CMCase activity was defined as the amount of enzyme producing 1 μmol of reducing sugar per min. Biomass concentration was indirectly measured by calculating the amount of total intracellular proteins, as described by Chen et al. [19]. CMCase and *p*NPCase activities were used to represent cellulase activity.

### RNA isolation and RT-qPCR

RNA extraction and RT-qPCR were performed as described by Chen et al. [19]. In brief, the FastRNA Pro Red Kit (MPbio, Irvine, CA, USA) was used to extract total RNA from mycelia. The TransScript One-Step gDNA Removal and cDNA Synthesis SuperMix (TransGen Biotech, Beijing, China) were used to synthesise cDNA from total RNA according to the manufacturer’s instructions. For RT-qPCR, the PerfectStart™ Green qPCR SuperMix (TransGen Biotech) was used to analyse the transcriptional levels of the main cellulase genes *cbh1* (encoding cellobiohydrolase I) and *egl1* (encoding endoglucanase I) using the 2^−ΔΔCt^ method. The sequences of the primers used in RT-qPCR are described in Additional File 14: Table S6. The transcriptional levels of *sar1* were measured for data normalisation [43].

### Determination of intracellular cAMP concentration

Cultures of liquid mycelia were harvested and frozen in liquid nitrogen. Intracellular cAMP extraction and determination were performed as previously described [44] with some modifications. In brief, the harvested mycelia were individually ground to a fine powder in liquid nitrogen and resuspended in phosphate-buffered saline (PBS, pH 7.0). After centrifugation, the supernatants were used to measure the intracellular concentration using the Microorganism cyclic adenosine monophosphate (cAMP) ELISA Kit (mlbio, Shanghai, China) according to the manufacturer’s protocol. The total protein content in each sample was determined using the Enhanced BCA Protein Assay Kit (Beyotime, Shanghai, China). The content of intracellular cAMP was expressed relative to the protein concentration in the same sample.

### Free cytosolic Ca^2+^ labelling and detection

Fluo-4 AM (Beyotime, Shanghai, China) was used as a Ca^2+^-specific probe to assess cytoplasmic Ca^2+^ concentrations according to the manufacturer’s protocol [26]. The mycelia were loaded with Fluo-4 AM (final concentration 5 μM) at 28 °C for 30 min and were washed three times with PBS (pH 5.0). The images of Fluo-4 AM labelled mycelia were viewed using an S Plan Fluor ELWD microscope at 20 × magnification with a 0.5 numerical aperture objective and digital sight camera on an Eclipse Ti inverted microscope system (Nikon, Tokyo, Japan) equipped with an FITC filter (420–490 nm band-pass excitation filter and 535 nm emission filter). The intensity of green fluorescence was quantified using the NIS-Elements F package software.

### Construction of plasmids and strains

To construct the *acy1* deletion mutant, the 799 bp upstream and 718 bp downstream fragments of *acy1* were generated from the genome of *T. reesei* QM6a using KOD-Plus-Neo (TOYOBO, Osaka, Japan). The primers used are listed in Additional File 14: Table S6. First, the upstream fragment was ligated into the *Pac*I and *Xba*I linearized LML2.1 [45] using the pEASY®-Uni Seamless Cloning and Assembly Kit (TransGen Biotech) to form pF*acy1*. Subsequently, the downstream fragment was inserted into *Swa*I-linearised pF*acy1* to form the binary vector pD*acy1* (Additional File 1: Figure S1) for the knockout of *acy1* in QM6a using *Agrobacterium*-mediated transformation [46]. The putative *acy1* disruption mutants (Δ*acy1*) generated by double crossover were verified by diagnostic PCR using the primers acy1-CF and acy1-CR and acy1-OF and acy1-OR (Additional File 1: Figure S1).

The correct *acy1* deletion strains should have a single copy of the 5- and 3-flanks of the *acy1* gene as that in the wild-type strain QM6a (acy1-F and acy1-R; Additional File 1: Figure S1a). The integration of single-copy DNA fragments into transformed clones was verified using RT-qPCR (Additional File 1: Figure S1a-b) following previous studies [47, 48]. Fungal genomic DNA was extracted using fungal DNA extraction kits (TianGen, Beijing, China). Genomic DNA was sonicated on ice in 30-s pulses using a Bioruptor (Diagenode s.a. BELGIUM) at low power to shear chromatin to an average length of 500–5000 bp. DNA was then used as a template for RT-qPCR. The genome of QM6a with a single copy of the 5- and 3-flanking *acy1* gene was used as a reference (acy1-F and acy1-R; Additional File 1: Figure S1a). The *sar1* gene was used as a reference gene, using the primers sar1-3/sar1-4. The primers used for the genes are listed in Additional File 14: Table S6.

The re-complementation cassettes of *acy1* were constructed by ligating the entire native *acy1* expression cassette, including the native promoter, *acy1* coding sequence, and terminator, to the *Swa*I site of LML2.1. The complementation vector pR*acy1* and the re-complementation mutants of *acy1* (R*acy1*) were generated using *Agrobacterium*-mediated transformation (Additional File 1: Figure S1). The re-complementation mutants of *plc-e* (R*plc-e*) were constructed similarly.

### Transcriptome analysis

The transcriptomes of *T. reesei* QM6a cultured alone or with 10 mM Mn^2+^ or 1% DMF [20] were compared. Conidia (2 × 10^6^) were cultivated at 28 °C (200 rpm) in 50 mL of MM (2% glucose as the sole carbon source) for 36–48 h. The mycelia were inoculated in 100 mL of freshly prepared MM containing 1% Avicel as the sole carbon source, with either no further addition of components or with the addition of 10 mM Mn^2+^ or 1% DMF grown for 36 h. Mycelia were then harvested from the cultures. All the mycelia of the WT (wild-type strain QM6a with no addition, prepared in duplicate), Mn (wild-type strain QM6a with 10 mM Mn^2+^ addition, prepared in duplicate), and DMF (wild-type strain QM6a with 1% DMF addition, prepared in duplicate) were pooled, resulting in six samples. The samples were sent to a company (mega genomics, Beijing, China) for preparation and RNA sequencing using a HiSeq X Ten apparatus (Illumina, San Diego, CA, USA). Two biological replicates of each condition were submitted for RNA sequencing. Differential expression analysis was performed using DESeq [49]. Genes whose adjusted *P* values were lower than 0.01, and whose log2-fold change values were lower than -1 or higher than 1 were selected as differentially expressed genes (DEGs).

Processing of individual samples was successful without a significant difference between the replicates (Additional File 15: Table S7). Following sequence quality control, the sequence reads were mapped to a *T. reesei* reference genome (genome.jgi.doe.gov/Trire2/Trire2.home.html) with 93.87 to 94.80% coverage (Additional File 15: Table S7) for bioinformatics analysis. There was a high correlation (Pearson correlation, r^2^ ≥ 0.898) between the two biological replicates of each condition used in the transcriptional analysis (Additional File 16: Figure S9).

The raw whole transcriptome shotgun sequencing data and the related protocols are available at the NCBI SRA web site (https://www.ncbi.nlm.nih.gov/sra/PRJNA510366) under accession number PRJNA510366.

### Statistical analysis

All experimental data shown in this paper were obtained from at least three independent samples with identical or similar results. The error bars indicate standard deviations (SDs) from the mean of triplicate determinations. Student’s *t-*test was used to compare two samples. Duncan’s multiple-range test was used for multiple comparisons. Within each set of experiments, *p* < 0.05 was considered to indicate a significant difference.

## Supplementary Information

**Additional file 1**: **Figure S1**. Construction and verification of Δacy1 strains. a. Schematic representation of the acy1 locus from the QM6a and Δacy1 strains. The region from +1 to +6649 bp relative to the translation start site of acy1 (grey box) was replaced with the hygromycin cassette (black box). The binding sites of primers on the genome of QM6a and Δacy1 are indicated by red arrows. The binding sites of primers on the hygromycin cassette are indicated by blue arrows. The expected sizes of the PCR verification products in the Δacy1 strains are indicated as numbers. Primer pairs (acy1-T1/ acy1-T2 and acy1-T3/acy1-T4) shown in purple were used to identify the copy number of integrated genes. b. Schematic representation of the Pacy1-acy1-Tacy1 cassette in Racy1 strains. The acy1 complementation cassette was constructed by ligating the whole gene sequence (including the 1500 bp promoter, coding sequence, and 500 bp terminator) into LML2.1. The primer pairs indicated were used in the verification of the expression cassette. c. PCR verification of Δacy1 strains. Lane M, DNA molecular mass maker Lane F, PCR amplification results using the acy1-CF/D70-4 pair. Lane R, PCR amplification results using the HG3.6/acy1-CR pair. Lane O, PCR amplification results using the acy1-OF/acy1-OR pair. Lane N, PCR amplification results using acy1-OF/ acy1-OR pair with water as a negative control. Δacy1-1, Δacy1-2, and Δacy1-3 represent three Δacy1 strains. QM6a as controls. d. PCR verification of Racy1 strains. Lane M, DNA molecular mass maker Lanes, PCR amplification results using the acy1-OF/acy1-OR pair with three Racy1 strains and Δacy1 strains as templates. e. Verification of copy numbers for Δacy1 transformants by qPCR. The genome of QM6a was used as a reference with a single copy. f. Growth rates of wild-type QM6a, Δacy1, and Racy1 strains in MM plates.

**Additional file 2**: **Figure S2**. Cellulase activities of T. reesei complementary strains Racy1 and Rplc-e under different addition conditions. pNPCase activity/mg biomass of T. reesei QM6a, Racy1 and Rplc-e strains supplemented with 10 mM Mn2+ or 1% DMF. T. reesei QM6a was used as the control. Values are the mean ± SD of the results from three independent experiments. Asterisks indicate significant differences from the control (*p < 0.05, Student’s t test).

**Additional file 3**: **Figure S3**. Effect of Mn2+/DMF/cAMP-induced cellulase overexpression. a and b. pNPCase activity/mg biomass (a) and CMCase activity/mg biomass (b) of T. reesei QM6a and Δacy1 strains supplemented with 5 mM dbcAMP, 10 mM Mn2+ or (and) 1% DMF. Values are the means ± SD of the results from three independent experiments. Asterisks indicate significant differences from the control (*p < 0.05, Student’s t test).

**Additional file 4**: **Figure S4**. Relative expression levels of cam (c), cna1 (d), and crz1 (e) in T. reesei QM6a and Δacy1 strains under different conditions. The QM6a and Δacy1 strains were cultured in MM with 2% glucose as the carbon source and then inoculated in fresh MM supplemented with 10 mM Mn2+, 1% DMF, 0.02 mΜ Forskolin, or 5 mM dbcAMP, with 1% Avicel as the carbon source. Cultures that were not supplemented with Mn2+, DMF, Forskolin, or dbcAMP were used as controls. QM6a cells cultured without the addition of Mn2+, DMF, Forskolin, or dbcAMP were used as the reference sample. Values are expressed as the mean ± SD of the results from three independent experiments. Asterisks indicate significant differences from the control (*p < 0.05, Student’s t test).

**Additional file 5**: **Figure S5**. Effect of cAMP/LaCl3-induced cellulase overexpression. a and b. pNPCase activity/mg biomass (a) and CMCase activity/mg biomass (b) of T. reesei QM6a strains supplemented with 5 mM dbcAMP and 5 mM LaCl3. Values are the means ± SD of the results from three independent experiments. Asterisks indicate significant differences from the control (*p < 0.05, Student’s t test).

**Additional file 6**: **Figure S6**. Volcano plot analysis of up- and downregulated genes of strains treated with or without 10 mM Mn2+ treatment. Volcano plot for differences in gene expression with no addition or 10 mM Mn2+ addition. Red dots indicate significantly upregulated genes, green dots indicate significantly downregulated genes, and grey dots indicate non-significantly different gene expression. The x-axis represents the logarithm of the differential multiple of a gene expression in two samples. The y-axis represents the negative log of a statistically significant change in gene expression. Control: parental strain QM6a without 10 mM Mn2+ treatment; Sample 1: parental strain QM6a with 10 mM Mn2+ treatment.

**Additional file 7**: **Table S1**. Genes that are significantly up- or down-regulated in T. reesei QM6a with 10 mM Mn2+ addition when compared with no addition.

**Additional file 8**: **Table S2**. Differential transcription of genes in response to Mn2+ and DMF as measured by RNA-sequencing.

**Additional file 9**: **Table S3**. The log2 fold changes of the major cellulose degradation-related genes under Mn2+/DMF addition conditions.

**Additional file 10**: **Table S4**. Whole transcriptome shotgun sequencing data and RT-qPCR verification of the plc-e gene under Mn2+ addition conditions.

**Additional file 11**: **Figure S7**. The growth rate related cellulase activity and transcription levels of cbh1 and egl1 after Mn2+ stimulation in wild-type QM6a and Δplc-e mutant.

**Additional file 12**: **Figure S8**. Relative expression levels of cam (c), cna1 (d), and crz1 (e) in T. reesei QM6a and Δplc-e strains. The QM6a and Δplc-e strains were cultured in MM with 2% glucose as the carbon source and then inoculated in fresh MM supplemented with 10 mM Mn2+, 1% DMF, 0.02 mΜ Forskolin, or 5 mM dbcAMP, with 1% Avicel as the carbon source. Cultures with no addition were used as controls. QM6a cells cultured with no addition were used as the reference sample. Values are expressed as the mean ± SD of the results from three independent experiments. Asterisks indicate significant differences from the control (*p < 0.05, Student’s t test).

**Additional file 13**: **Table S5**. The log2 fold changes of the putative GPCRs genes under Mn2+/DMF addition conditions.

**Additional file 14**: **Table S6**. Primers used in this study.

**Additional file 15**: **Table S7**. Sequencing statistics for whole transcriptome shotgun sequencing results from this study.

**Additional file 16**: **Figure S9**. Biological replicates used for whole-transcriptome shotgun sequencing analysis. Graphs representing the Pearson correlation between biological replicates of each sample. A high Pearson correlation was obtained, demonstrating the reliability of whole transcriptome shotgun sequencing analysis (r2 ≥ 0.898). The x-axis and y-axis correspond to the gene expression in different treatments (e.g., experiment or control) after conversion by log2(FPKM+1).

## Data Availability

All data generated or analysed during this study are included in this published article [and its supplementary information files].
