# Supplementary figures and images for: cAMP activates calcium signalling via phospholipase C to regulate cellulase production in the filamentous fungus Trichoderma reesei
Source: Biotechnol Biofuels. 2021 Mar 8;14:62. doi: 10.1186/s13068-021-01914-0 (PMC7941909; doi:10.1186/s13068-021-01914-0)

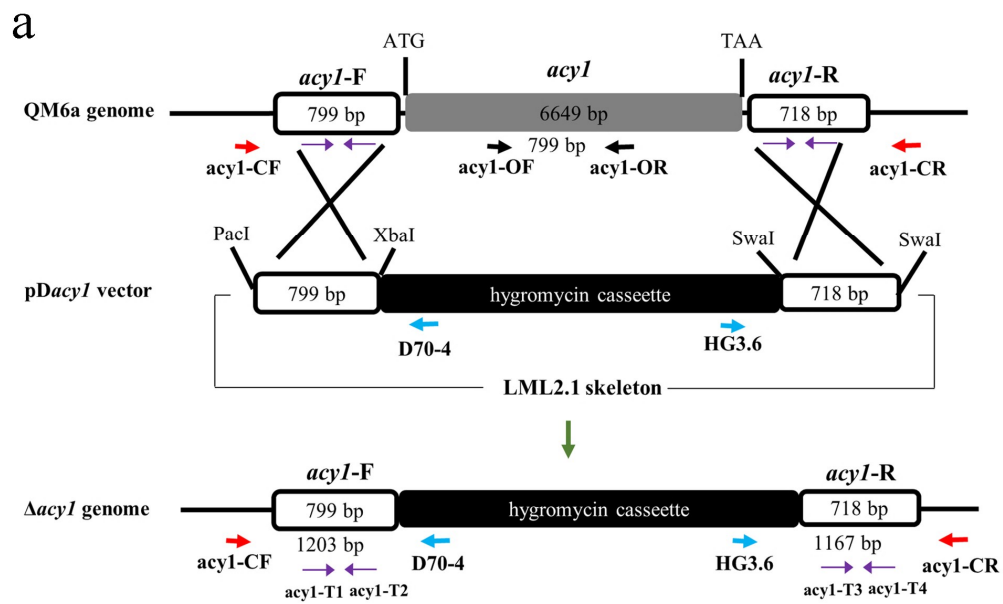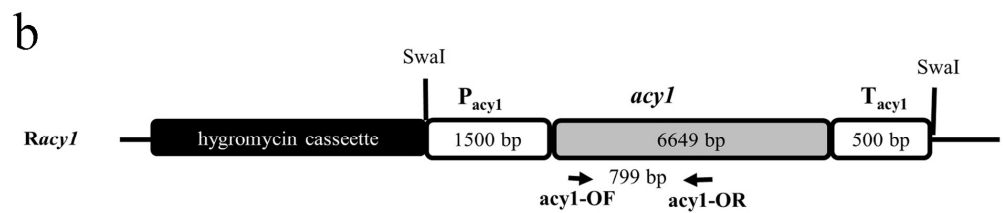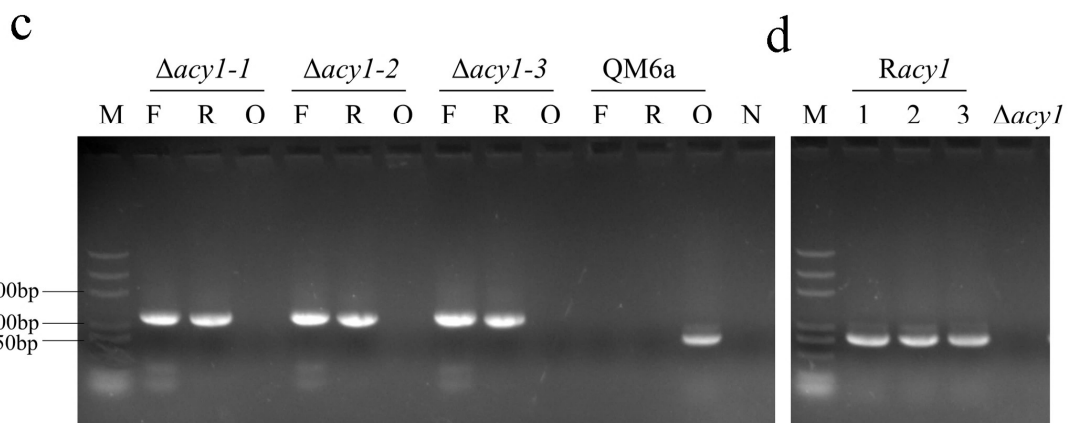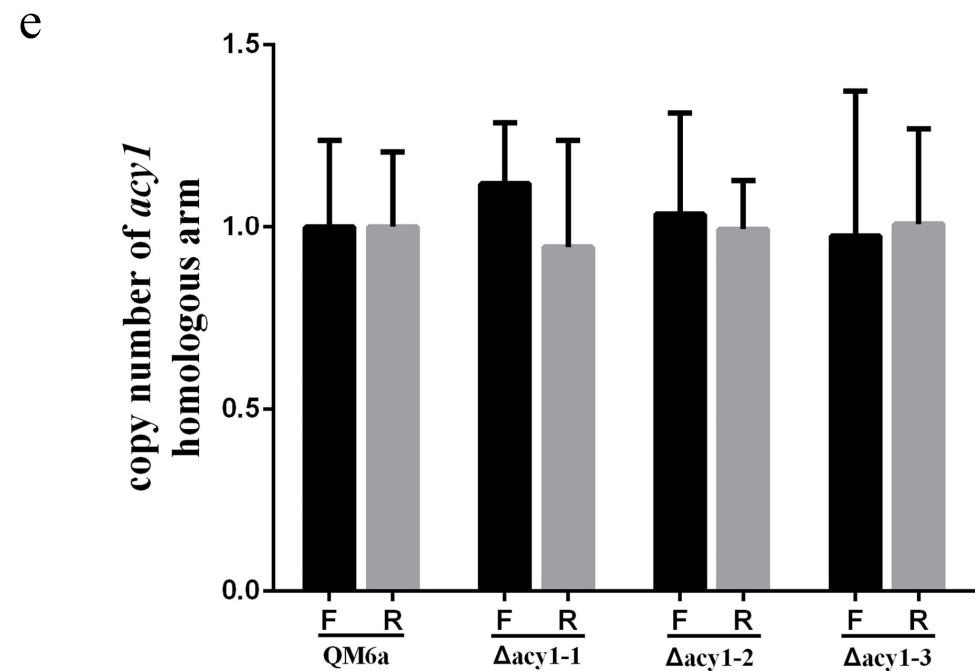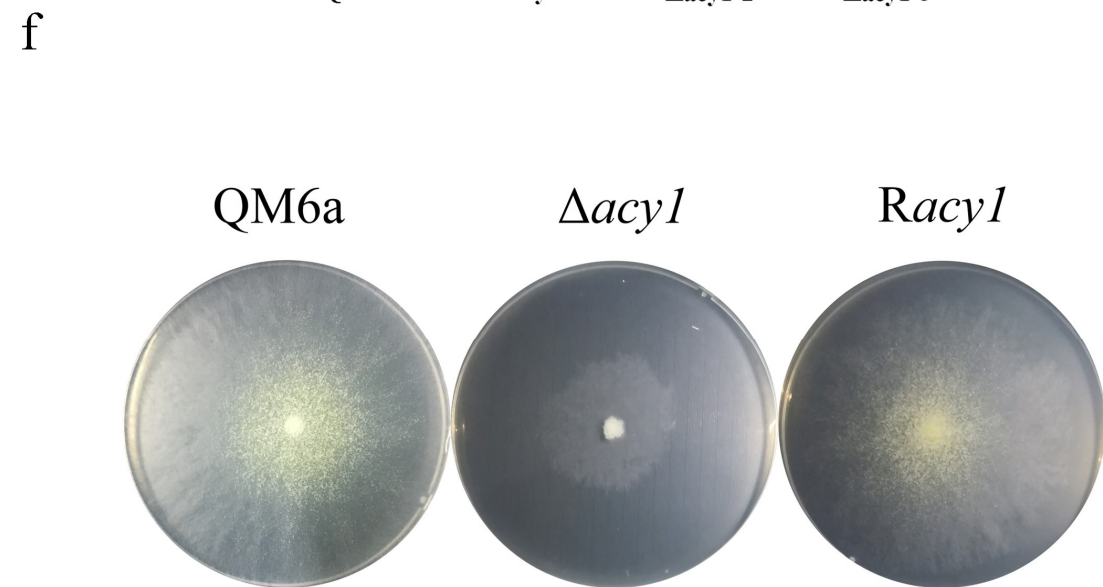

Supplement: Supplementary file 1 — Additional file 1: Figure S1. Construction and verification of Δacy1 strains. a. Schematic representation of the acy1 locus from the QM6a and Δacy1 strains. The region from +1 to +6649 bp relative to the translation start site of acy1 (grey box) was replaced with the hygromycin cassette (black box). The binding sites of primers on the genome of QM6a and Δacy1 are indicated by red arrows. The binding sites of primers on the hygromycin cassette are indicated by blue arrows. The expected sizes of the PCR verification products in the Δacy1 strains are indicated as numbers. Primer pairs (acy1-T1/ acy1-T2 and acy1-T3/acy1-T4) shown in purple were used to identify the copy number of integrated genes. b. Schematic representation of the Pacy1-acy1-Tacy1 cassette in Racy1 strains. The acy1 complementation cassette was constructed by ligating the whole gene sequence (including the 1500 bp promoter, coding sequence, and 500 bp terminator) into LML2.1. The primer pairs indicated were used in the verification of the expression cassette. c. PCR verification of Δacy1 strains. Lane M, DNA molecular mass maker Lane F, PCR amplification results using the acy1-CF/D70-4 pair. Lane R, PCR amplification results using the HG3.6/acy1-CR pair. Lane O, PCR amplification results using the acy1-OF/acy1-OR pair. Lane N, PCR amplification results using acy1-OF/ acy1-OR pair with water as a negative control. Δacy1-1, Δacy1-2, and Δacy1-3 represent three Δacy1 strains. QM6a as controls. d. PCR verification of Racy1 strains. Lane M, DNA molecular mass maker Lanes, PCR amplification results using the acy1-OF/acy1-OR pair with three Racy1 strains and Δacy1 strains as templates. e. Verification of copy numbers for Δacy1 transformants by qPCR. The genome of QM6a was used as a reference with a single copy. f. Growth rates of wild-type QM6a, Δacy1, and Racy1 strains in MM plates. [file 13068_2021_1914_MOESM1_ESM.pdf]

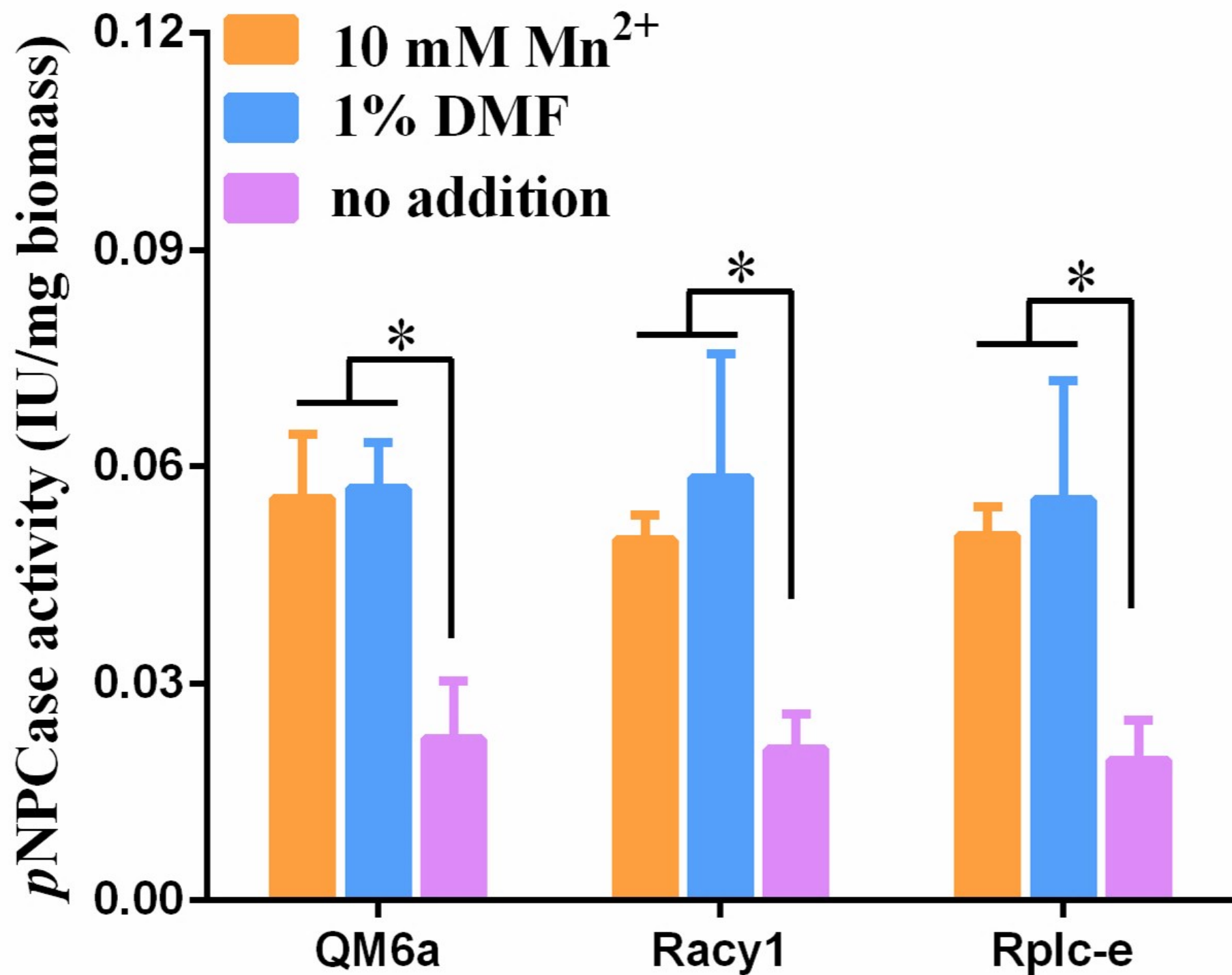

Supplement: Supplementary file 2 — Additional file 2: Figure S2. Cellulase activities of T. reesei complementary strains Racy1 and Rplc-e under different addition conditions. pNPCase activity/mg biomass of T. reesei QM6a, Racy1 and Rplc-e strains supplemented with 10 mM Mn2+ or 1% DMF. T. reesei QM6a was used as the control. Values are the mean ± SD of the results from three independent experiments. Asterisks indicate significant differences from the control (*p < 0.05, Student’s t test). [file 13068_2021_1914_MOESM2_ESM.pdf]

a

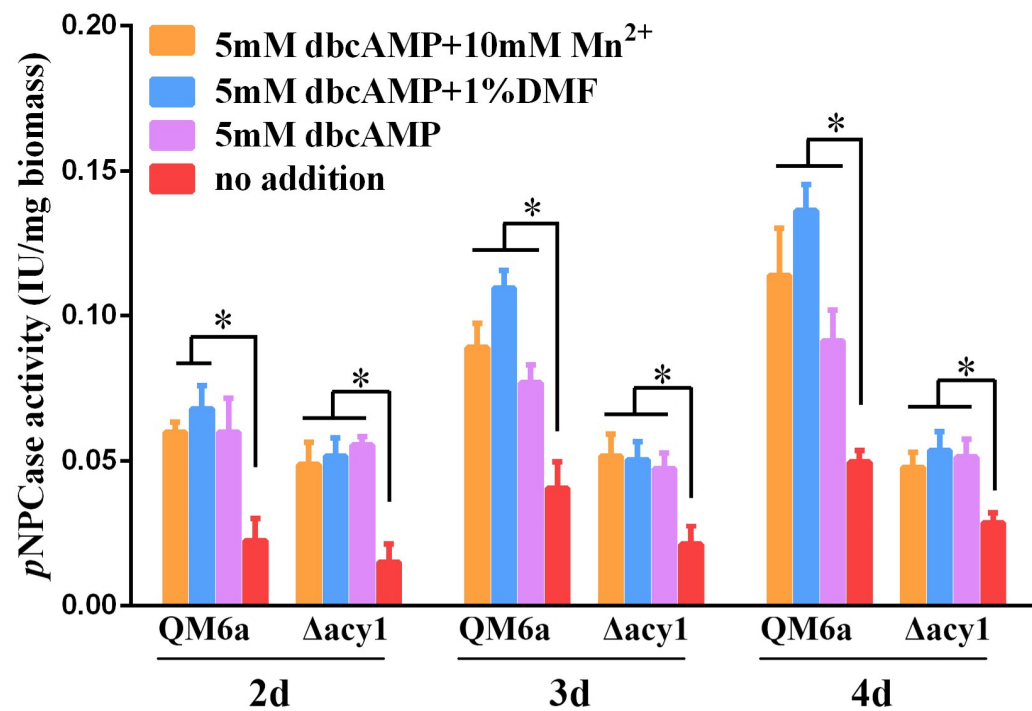

b

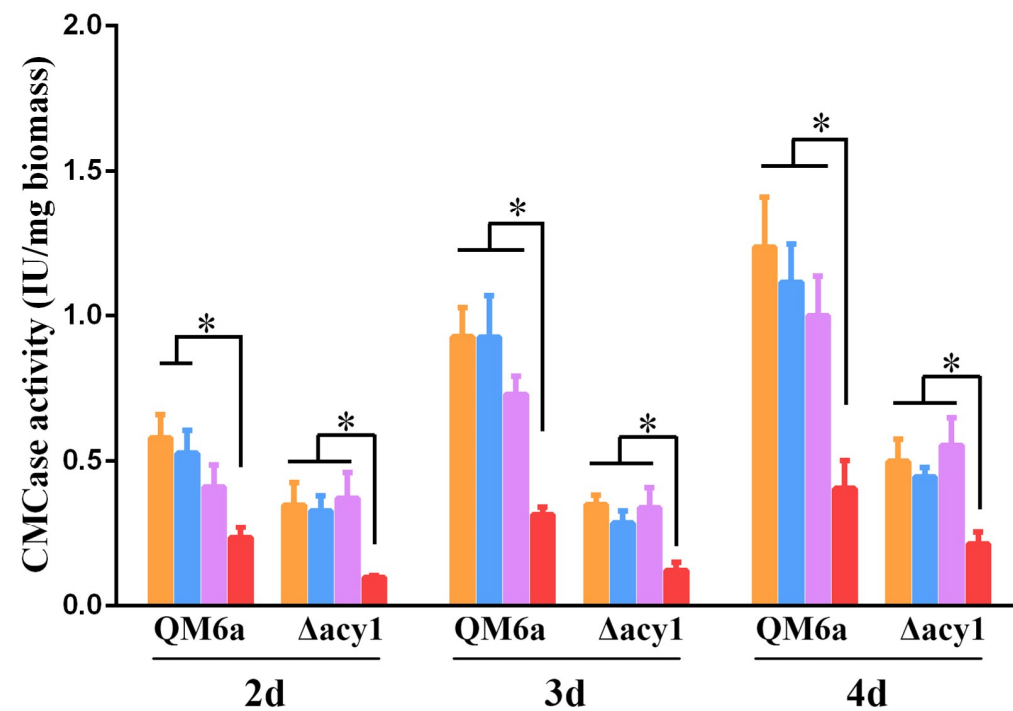

Supplement: Supplementary file 3 — Additional file 3: Figure S3. Effect of Mn2+/DMF/cAMP-induced cellulase overexpression. a and b. pNPCase activity/mg biomass (a) and CMCase activity/mg biomass (b) of T. reesei QM6a and Δacy1 strains supplemented with 5 mM dbcAMP, 10 mM Mn2+ or (and) 1% DMF. Values are the means ± SD of the results from three independent experiments. Asterisks indicate significant differences from the control (*p < 0.05, Student’s t test). [file 13068_2021_1914_MOESM3_ESM.pdf]

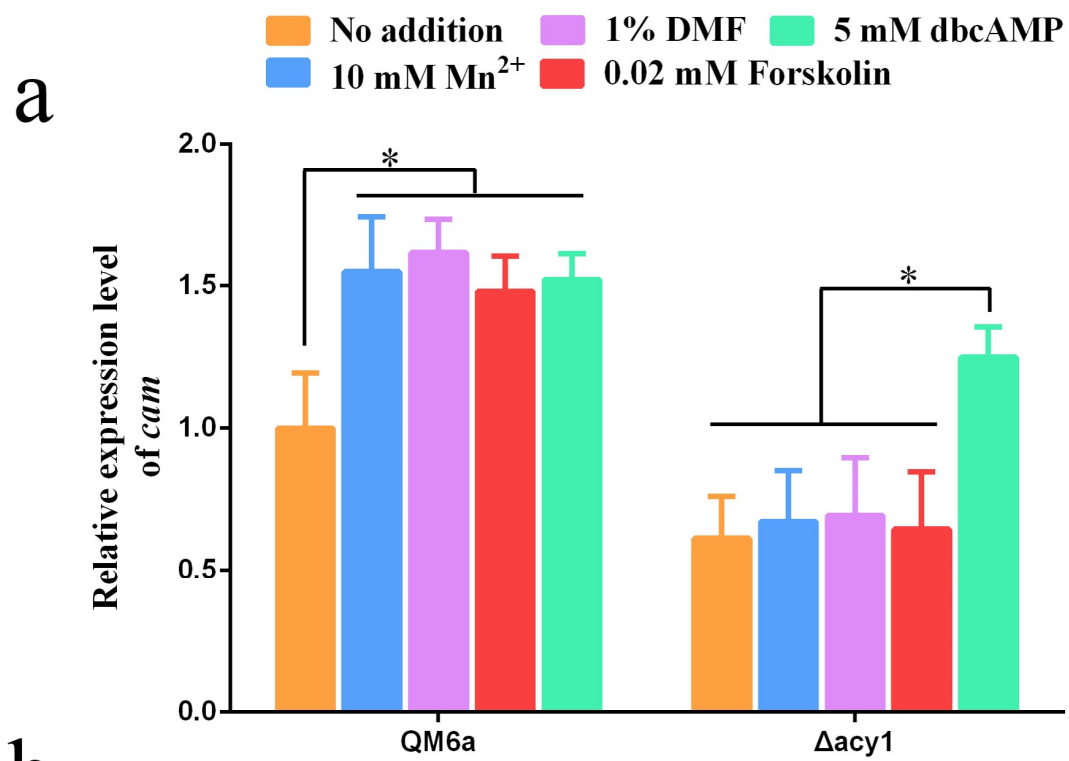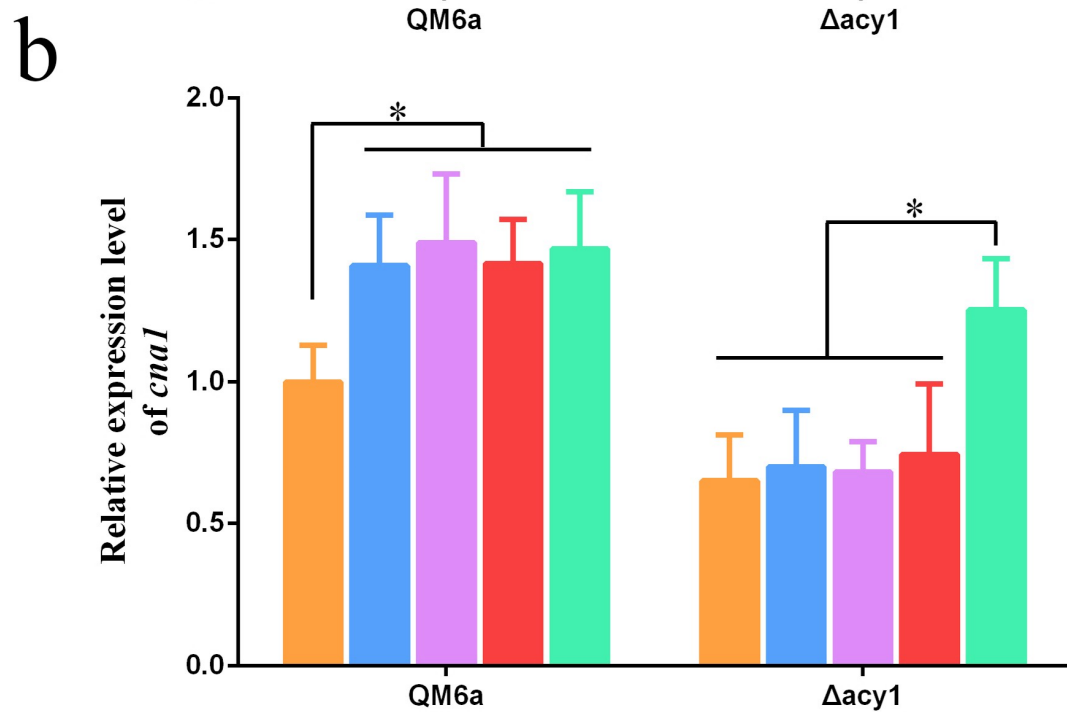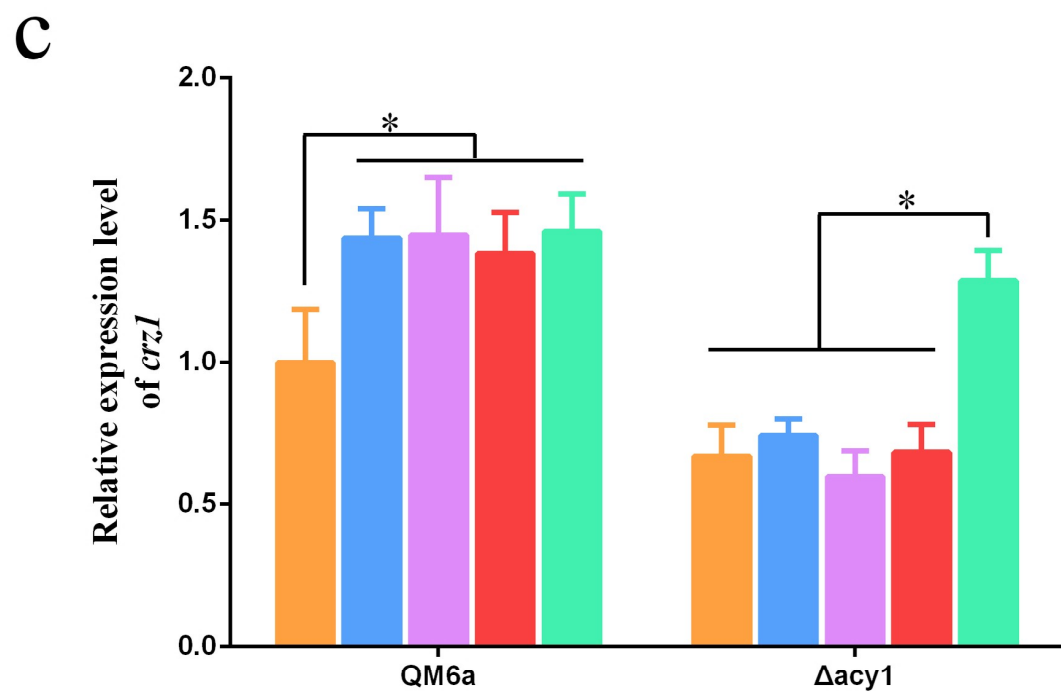

Supplement: Supplementary file 4 — Additional file 4: Figure S4. Relative expression levels of cam (c), cna1 (d), and crz1 (e) in T. reesei QM6a and Δacy1 strains under different conditions. The QM6a and Δacy1 strains were cultured in MM with 2% glucose as the carbon source and then inoculated in fresh MM supplemented with 10 mM Mn2+, 1% DMF, 0.02 mΜ Forskolin, or 5 mM dbcAMP, with 1% Avicel as the carbon source. Cultures that were not supplemented with Mn2+, DMF, Forskolin, or dbcAMP were used as controls. QM6a cells cultured without the addition of Mn2+, DMF, Forskolin, or dbcAMP were used as the reference sample. Values are expressed as the mean ± SD of the results from three independent experiments. Asterisks indicate significant differences from the control (*p < 0.05, Student’s t test). [file 13068_2021_1914_MOESM4_ESM.pdf]

**a**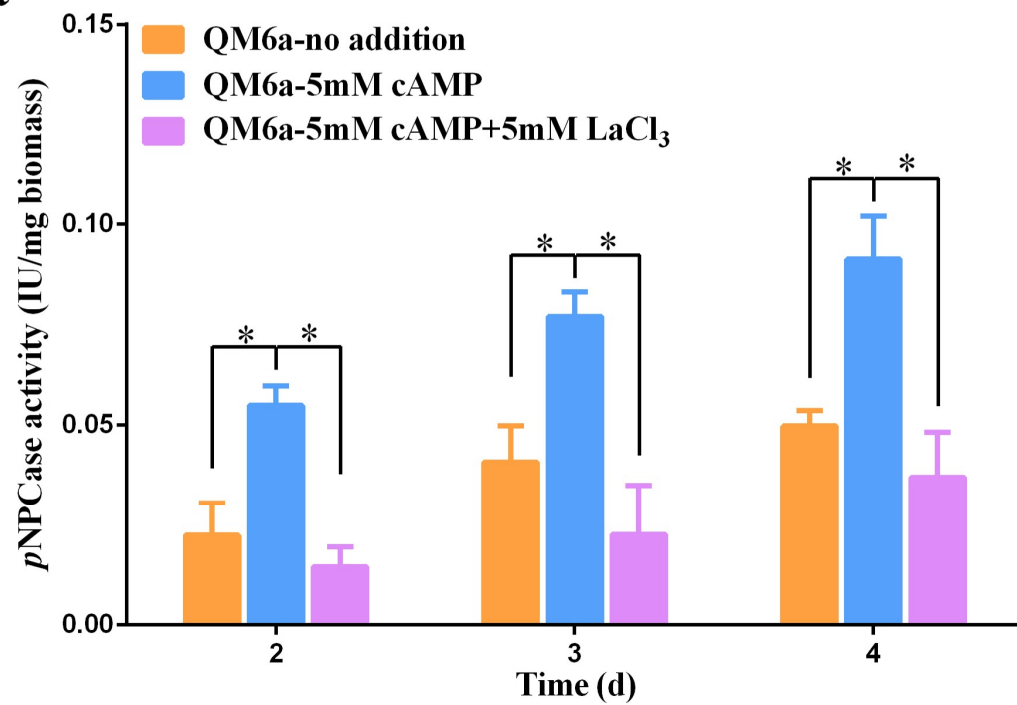**b**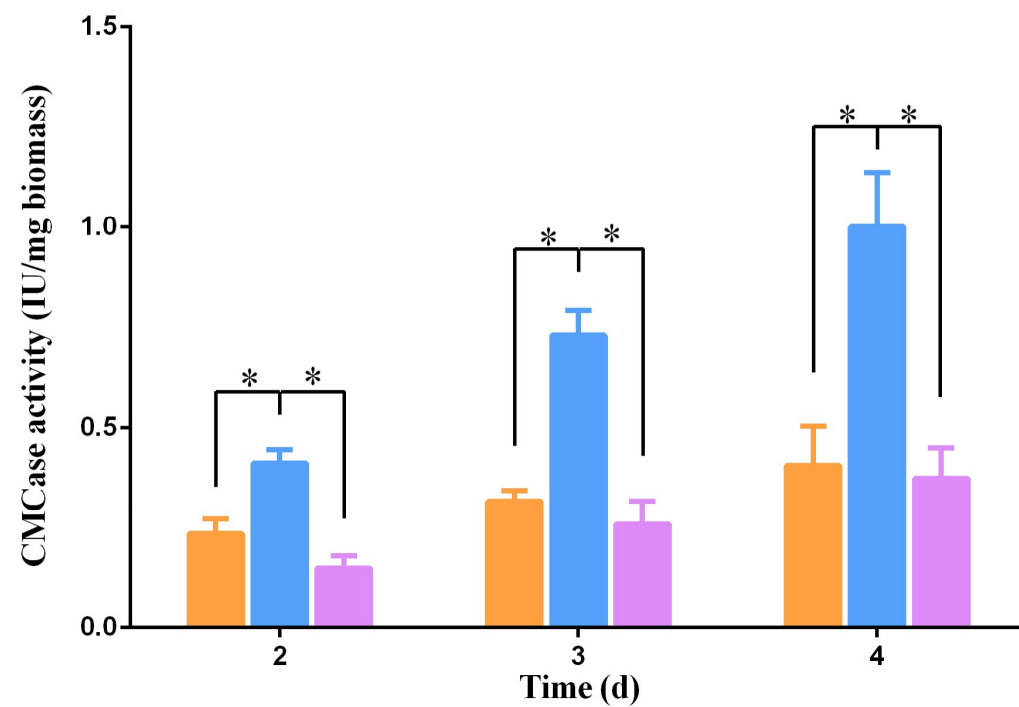

Supplement: Supplementary file 5 — Additional file 5: Figure S5. Effect of cAMP/LaCl3-induced cellulase overexpression. a and b. pNPCase activity/mg biomass (a) and CMCase activity/mg biomass (b) of T. reesei QM6a strains supplemented with 5 mM dbcAMP and 5 mM LaCl3. Values are the means ± SD of the results from three independent experiments. Asterisks indicate significant differences from the control (*p < 0.05, Student’s t test). [file 13068_2021_1914_MOESM5_ESM.pdf]

Volcano plot(control\_vs\_sample1)

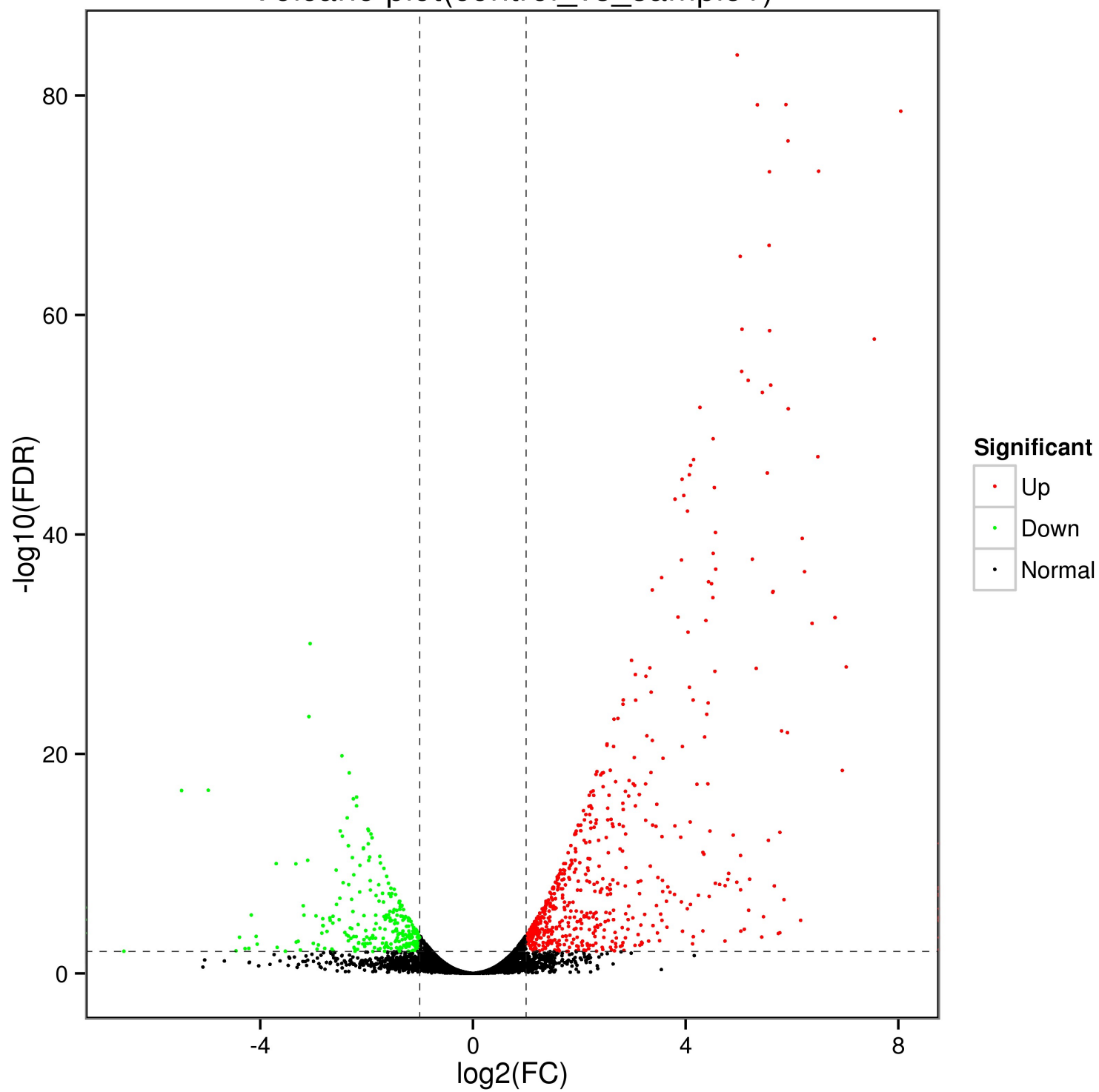

Supplement: Supplementary file 6 — Additional file 6: Figure S6. Volcano plot analysis of up- and downregulated genes of strains treated with or without 10 mM Mn2+ treatment. Volcano plot for differences in gene expression with no addition or 10 mM Mn2+ addition. Red dots indicate significantly upregulated genes, green dots indicate significantly downregulated genes, and grey dots indicate non-significantly different gene expression. The x-axis represents the logarithm of the differential multiple of a gene expression in two samples. The y-axis represents the negative log of a statistically significant change in gene expression. Control: parental strain QM6a without 10 mM Mn2+ treatment; Sample 1: parental strain QM6a with 10 mM Mn2+ treatment. [file 13068_2021_1914_MOESM6_ESM.pdf]

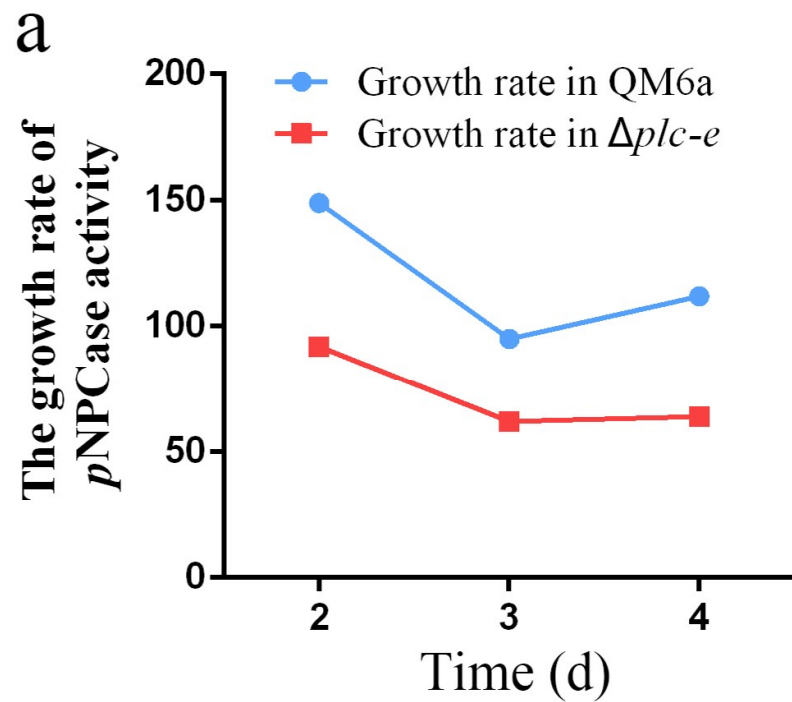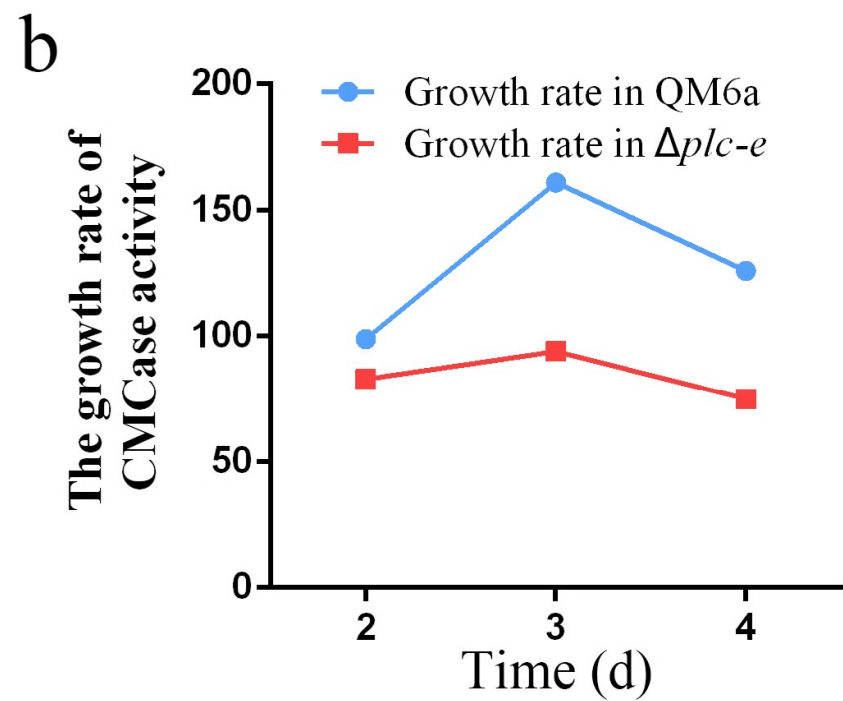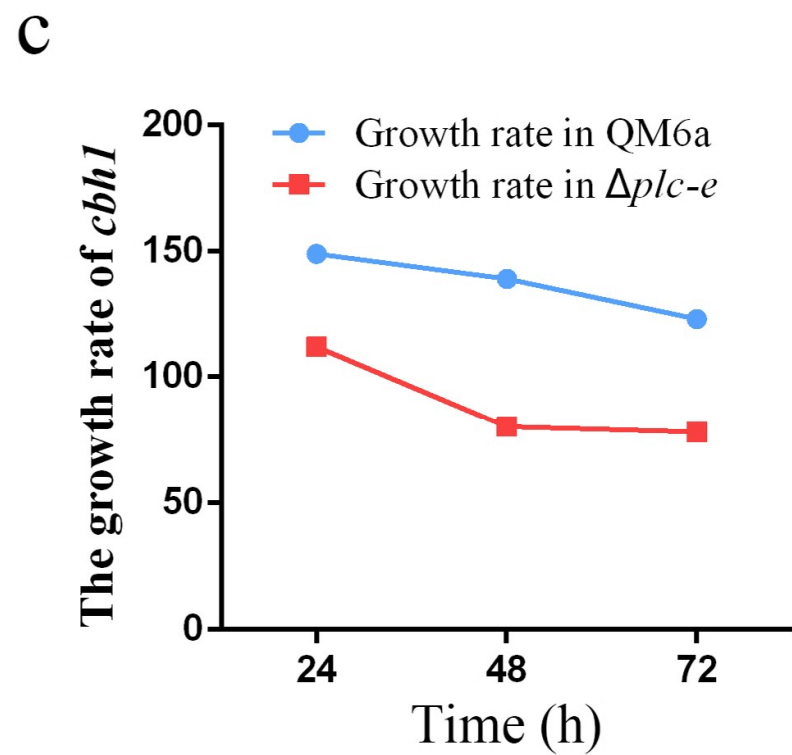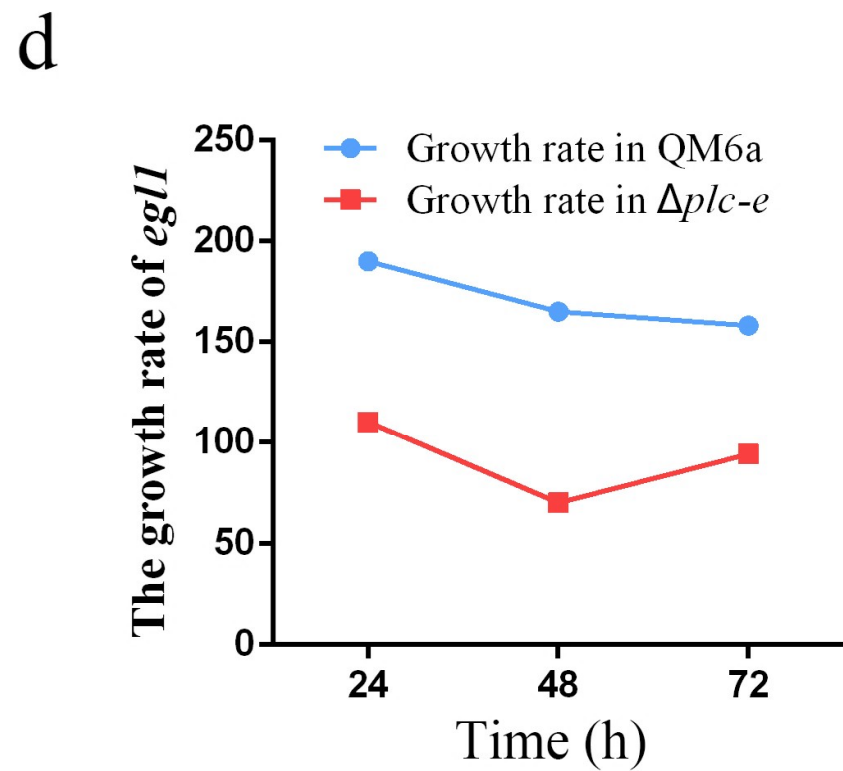

Supplement: Supplementary file 11 — Additional file 11: Figure S7. The growth rate related cellulase activity and transcription levels of cbh1 and egl1 after Mn2+ stimulation in wild-type QM6a and Δplc-e mutant. [file 13068_2021_1914_MOESM11_ESM.pdf]

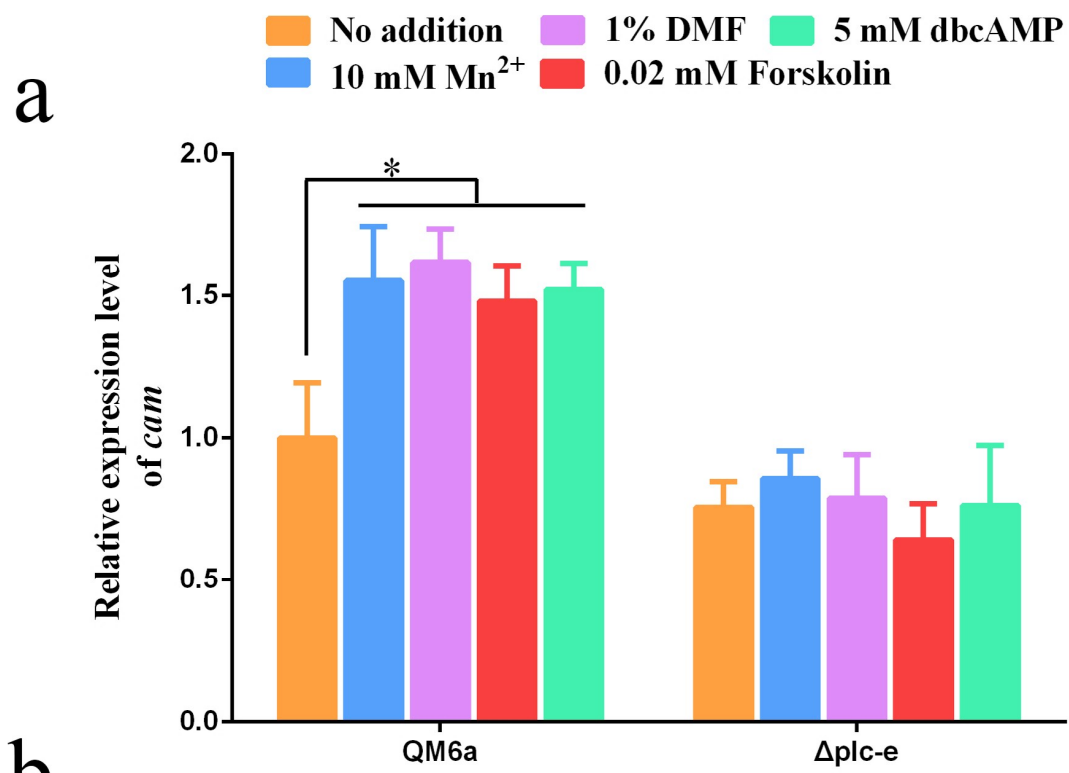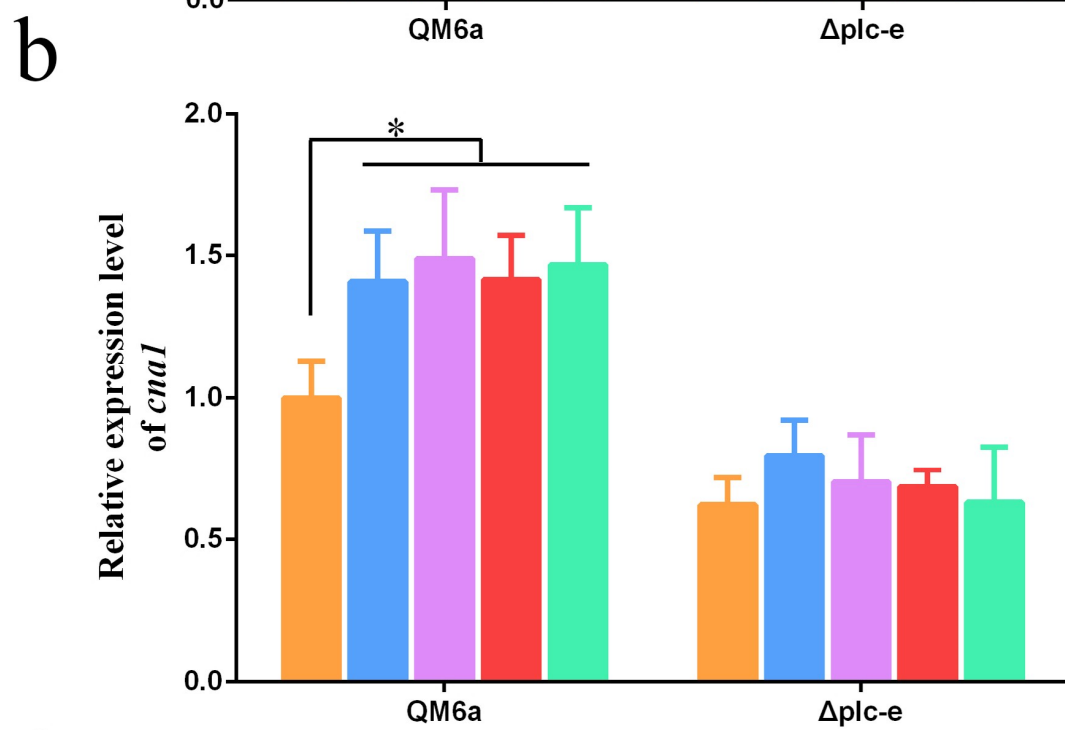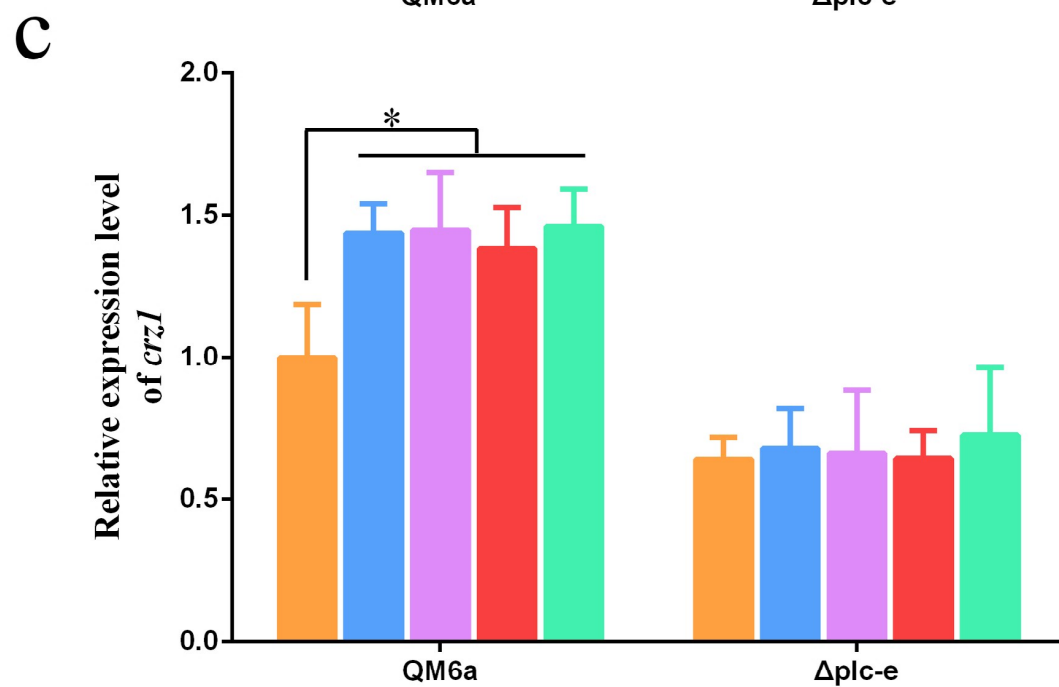

Supplement: Supplementary file 12 — Additional file 12: Figure S8. Relative expression levels of cam (c), cna1 (d), and crz1 (e) in T. reesei QM6a and Δplc-e strains. The QM6a and Δplc-e strains were cultured in MM with 2% glucose as the carbon source and then inoculated in fresh MM supplemented with 10 mM Mn2+, 1% DMF, 0.02 mΜ Forskolin, or 5 mM dbcAMP, with 1% Avicel as the carbon source. Cultures with no addition were used as controls. QM6a cells cultured with no addition were used as the reference sample. Values are expressed as the mean ± SD of the results from three independent experiments. Asterisks indicate significant differences from the control (*p < 0.05, Student’s t test). [file 13068_2021_1914_MOESM12_ESM.pdf]

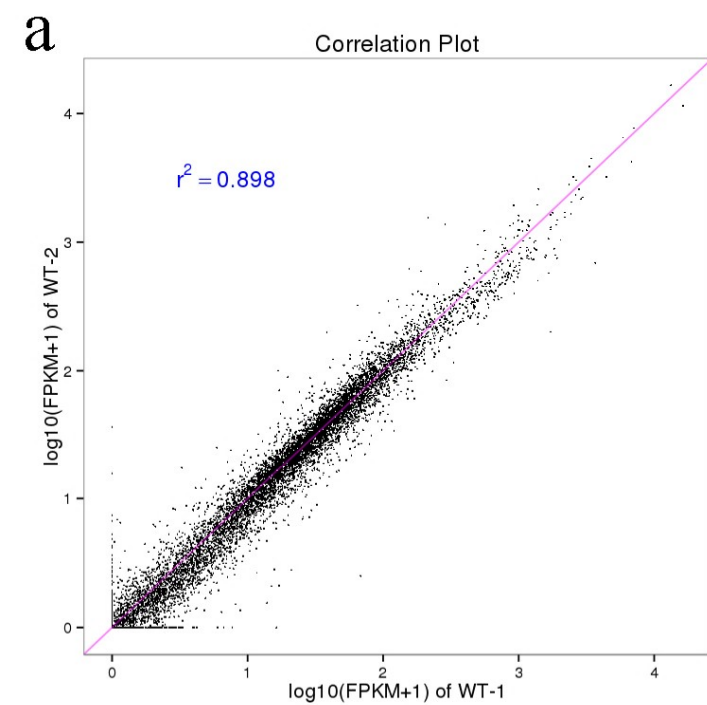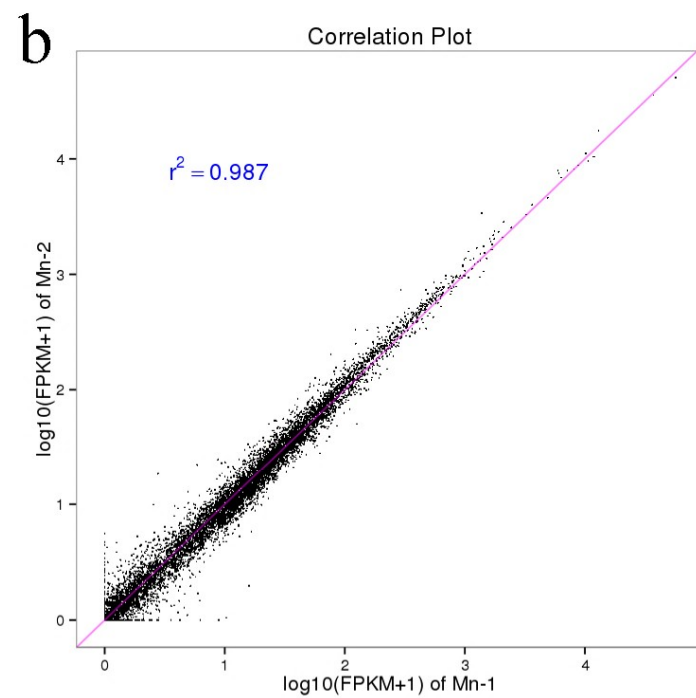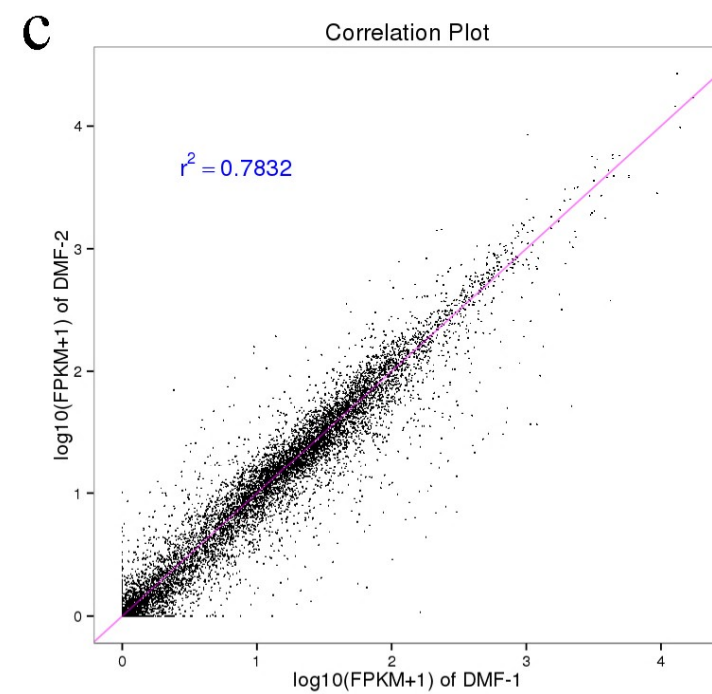

Supplement: Supplementary file 16 — Additional file 16: Figure S9. Biological replicates used for whole-transcriptome shotgun sequencing analysis. Graphs representing the Pearson correlation between biological replicates of each sample. A high Pearson correlation was obtained, demonstrating the reliability of whole transcriptome shotgun sequencing analysis (r2 ≥ 0.898). The x-axis and y-axis correspond to the gene expression in different treatments (e.g., experiment or control) after conversion by log2(FPKM+1). [file 13068_2021_1914_MOESM16_ESM.pdf]
