# Supplementary material for: cAMP activates calcium signalling via phospholipase C to regulate cellulase production in the filamentous fungus Trichoderma reesei
Source: Biotechnol Biofuels. 2021 Mar 8;14:62. doi: 10.1186/s13068-021-01914-0 (PMC7941909; doi:10.1186/s13068-021-01914-0)
Supplement: Supplementary file 8 — Additional file 8: Table S2. Differential transcription of genes in response to Mn2+ and DMF as measured by RNA-sequencing. [file 13068_2021_1914_MOESM8_ESM.docx]

**Supplementary Table S2. Differential transcription of genes in response to Mn^2+^ and DMF as measured by RNA-sequencing.**

| #ID | | **Mn vs WT** | | **DMF vs WT** | |
| --- | --- | --- | --- | --- | --- |
|  |  | log_2_FC | regulated | log_2_FC | regulated |
| 46882 | estExt_Genewise1.C_60207 | 1.805234377 | up | 2.29263597 | up |
| 51893 | estExt_Genewise1.C_260107 | 3.326258234 | up | 3.071488186 | up |
| 80980 | estExt_GeneWisePlus.C_210298 | 5.57757007 | up | 5.02901112 | up |
| 121735 | estExt_fgenesh5_pg.C_90015 | 2.45281182 | up | 2.72764874 | up |
| 43974 | estExt_Genewise1.C_10758 | 2.279496999 | up | 2.042235974 | up |
| 120697 | estExt_fgenesh5_pg.C_40259 | 4.418491392 | up | 4.081059612 | up |
| 105171 | fgenesh5_pg.C_scaffold_4000549 | 5.771133808 | up | 5.759310667 | up |
| 108143 | fgenesh5_pg.C_scaffold_11000116 | 5.02677065 | up | 6.376365258 | up |
| 44362 | estExt_Genewise1.C_11536 | 6.486884741 | up | 7.745549074 | up |
| 123079 | estExt_fgenesh5_pg.C_180001 | 6.378287186 | up | 4.809720184 | up |
| 106315 | fgenesh5_pg.C_scaffold_7000116 | 2.982322207 | up | 1.695315668 | up |
| 44967 | estExt_Genewise1.C_20662 | 2.808720475 | up | 2.45322614 | up |
| 79741 | estExt_GeneWisePlus.C_150085 | 1.680028229 | up | 1.73539053 | up |
| 72567 | kg2.C_scaffold_3000002 | 5.349191929 | up | 3.553738877 | up |
| 21960 | estExt_fgenesh1_pm.C_50195 | 3.964804412 | up | 2.106057918 | up |
| 112140 | fgenesh5_pg.C_scaffold_33000038 | 5.053314934 | up | 2.856726383 | up |
| 82032 | estExt_GeneWisePlus.C_290155 | 2.914887713 | up | 3.358555779 | up |
| 109806 | fgenesh5_pg.C_scaffold_17000136 | 2.582845401 | up | 1.895236314 | up |
| 72704 | kg2.C_scaffold_30000003 | 2.321735468 | up | 2.132062561 | up |
| 108144 | fgenesh5_pg.C_scaffold_11000117 | 4.212571847 | up | 6.03495665 | up |
| 49976 | estExt_Genewise1.C_150113 | 5.535902472 | up | 3.783179 | up |
| 120504 | estExt_fgenesh5_pg.C_30502 | 4.430590189 | up | 2.973816339 | up |
| 55319 | e_gw1.2.255.1 | 1.755509147 | up | 4.203549184 | up |
| 103049 | fgenesh5_pg.C_scaffold_1000682 | 5.915077842 | up | 4.092421622 | up |
| 108357 | fgenesh5_pg.C_scaffold_11000330 | 3.055426472 | up | 2.735300372 | up |
| 123616 | estExt_fgenesh5_pg.C_240056 | 4.034667015 | up | 2.469957497 | up |
| 123989 | estExt_fgenesh5_pg.C_290113 | 4.970791054 | up | 3.159321793 | up |
| 123797 | estExt_fgenesh5_pg.C_260111 | 3.479148637 | up | 2.56127918 | up |
| 55351 | e_gw1.2.556.1 | 1.495760293 | up | 2.080093875 | up |
| 80639 | estExt_GeneWisePlus.C_190288 | 4.540118346 | up | 2.636036898 | up |
| 56684 | e_gw1.3.731.1 | 4.5173063 | up | 3.25582471 | up |
| 53053 | e_gw1.1.1931.1 | 1.95913633 | up | 2.91156719 | up |
| 81383 | estExt_GeneWisePlus.C_240187 | 3.372313788 | up | 3.168352849 | up |
| 123940 | estExt_fgenesh5_pg.C_280132 | 5.925023609 | up | 3.720140057 | up |
| 123992 | estExt_fgenesh5_pg.C_290118 | 4.486557614 | up | 3.352034769 | up |
| 120229 | estExt_fgenesh5_pg.C_20480 | 5.637895787 | up | 3.978442726 | up |
| 76210 | estExt_GeneWisePlus.C_50020 | 4.424627603 | up | 3.157560027 | up |
| 122081 | estExt_fgenesh5_pg.C_100226 | 5.176324049 | up | 3.823482848 | up |
| 120311 | estExt_fgenesh5_pg.C_30012 | 3.800776803 | up | 2.391487063 | up |
| 124198 | estExt_fgenesh5_pg.C_380025 | 2.868055482 | up | 3.342627123 | up |
| 54675 | e_gw1.1.1404.1 | 3.059997578 | up | 2.362656776 | up |
| 55272 | e_gw1.2.839.1 | 3.373957632 | up | 3.997814249 | up |
| 120312 | estExt_fgenesh5_pg.C_30013 | 5.442794651 | up | 4.046207124 | up |
| 108145 | fgenesh5_pg.C_scaffold_11000118 | 6.946745945 | up | 8.546901368 | up |
| 21716 | estExt_fgenesh1_pm.C_40045 | 1.676816667 | up | 2.245041822 | up |
| 69276 | e_gw1.28.211.1 | 5.929913659 | up | 3.842713056 | up |
| 76172 | estExt_GeneWisePlus.C_40970 | 2.828024312 | up | 1.74249402 | up |
| 106171 | fgenesh5_pg.C_scaffold_6000397 | 4.559305583 | up | 3.99044199 | up |
| 22560 | estExt_fgenesh1_pm.C_120015 | 2.559560922 | up | 1.915192499 | up |
| 73536 | estExt_GeneWisePlus.C_10096 | 5.57044843 | up | 3.754450906 | up |
| 104295 | fgenesh5_pg.C_scaffold_3000250 | 5.57044843 | up | 3.754450906 | up |
| 104227 | fgenesh5_pg.C_scaffold_3000182 | 5.328328775 | up | 4.381238387 | up |
| 111681 | fgenesh5_pg.C_scaffold_28000094 | 2.326208023 | up | 2.641386014 | up |
| 121682 | estExt_fgenesh5_pg.C_80351 | 1.643823107 | up | 1.982955145 | up |
| 58990 | e_gw1.5.363.1 | Inf | up | Inf | up |
| 29642 | gw1.3.294.1 | Inf | up | Inf | up |
| 77577 | estExt_GeneWisePlus.C_80473 | -2.147657171 | down | -3.502804364 | down |
| 123468 | estExt_fgenesh5_pg.C_220056 | -1.95966343 | down | -1.694100365 | down |
| 123260 | estExt_fgenesh5_pg.C_190147 | -3.330490643 | down | -3.789712033 | down |
| 82626 | estExt_GeneWisePlus.C_460020 | -1.246872999 | down | -2.215804623 | down |
| 104322 | fgenesh5_pg.C_scaffold_3000277 | -1.337302748 | down | -1.829004939 | down |
| 68028 | e_gw1.23.216.1 | -1.821990133 | down | -2.25183115 | down |
| 59778 | e_gw1.6.122.1 | -1.617533844 | down | -2.86741049 | down |
