# Supplementary material for: cAMP activates calcium signalling via phospholipase C to regulate cellulase production in the filamentous fungus Trichoderma reesei
Source: Biotechnol Biofuels. 2021 Mar 8;14:62. doi: 10.1186/s13068-021-01914-0 (PMC7941909; doi:10.1186/s13068-021-01914-0)
Supplement: Supplementary file 9 — Additional file 9: Table S3. The log2 fold changes of the major cellulose degradation-related genes under Mn2+/DMF addition conditions. [file 13068_2021_1914_MOESM9_ESM.docx]

**Supplementary Table S3. The log_2_ fold changes of the major cellulose degradation-related genes under Mn^2+^/DMF addition conditions.**

| **Gene ID** | **Annotation** | **log_2_ fold change**  **(Mn^b^ vs** **WT^a^)** | **log_2_ fold change**  **(DMF^c^** **vs** **WT^a^)** | | **Regulate** |
| --- | --- | --- | --- | --- | --- |
| Trire2_123989 | cellobiohydeolase CBH1/Cel7a | 4.9707910542 | | 3.159321793 | up |
| Trire2_72567 | cellobiohydeolase CBH2/Cel6a | 5.3491919286 | | 3.553738877 | up |
| Trire2_122081  Trire2_120312  Trire2_49976 | endoglucanase EGL1/Cel7b  endoglucanase EGL2/Cel5a  endoglucanase EGL5/Cel45a | 5.1763240486  5.4427946512  5.5359024722 | | 3.823482848  4.046207124  3.783179000 | up  up  up |
| Trire2_121735 | beta-glucosidase Cel3b | 2.4528118199 | | 2.727648740 | up |
| Trire2_120229 | endo-1,4-beta-xylanase XYNIII | 5.6378957866 | | 3.978442725 | up |
| Trire2_123992 | Swollenin | 4.4865576139 | | 3.352034769 | up |
| Trire2_69276 | Cellulosome enzyme | 5.9299136587 | | 3.842713056 | up |
| Trire2_76210 | Glycoside hydrolase | 4.4246276032 | | 3.157560026 | up |
| Trire2_123940 | cellulose-binding protein CIP2 | 5.9250236094 | | 3.7201400571 | up |

^a^ WT, the gene expression level the wild-type strain QM6a with no addition.

^b^ Mn, the gene expression level in the wild-type strain QM6a with 10 mM Mn^2+^ addition.

^c^ DMF, the gene expression level in the wild-type strain QM6a with 1% DMF addition.
