# Supplementary material for: cAMP activates calcium signalling via phospholipase C to regulate cellulase production in the filamentous fungus Trichoderma reesei
Source: Biotechnol Biofuels. 2021 Mar 8;14:62. doi: 10.1186/s13068-021-01914-0 (PMC7941909; doi:10.1186/s13068-021-01914-0)
Supplement: Supplementary file 10 — Additional file 10: Table S4. Whole transcriptome shotgun sequencing data and RT-qPCR verification of the plc-e gene under Mn2+ addition conditions. [file 13068_2021_1914_MOESM10_ESM.docx]

**Supplementary Table S4. Whole transcriptome shotgun sequencing data and RT-qPCR verification of the *plc-e* gene under Mn^2+^ addition conditions.**

| **Gene ID** | **Gene name** | **Gene product** | **log_2_ fold change**  **(Mn^b^ vs** **WT^a^)** | **log_2_ fold change**  **(Mn vs** **WT)** |
| --- | --- | --- | --- | --- |
|  |  |  | **RNA-seq^c^** | **RT-qPCR^d^** |
| Trire2:21960 | *plc-e* | phospholipase C | 3.96 | 4.08 |

^a^ WT, the gene expression level the wild-type strain QM6a with no addition.

^b^ Mn, the gene expression level in the wild-type strain QM6a with 10 mM Mn^2+^ addition.

^c^ Results from FPKM values shown in the RNA-Seq data.

^d^ Results of relative expression levels obtained by RT-qPCR assays. The values are means of the results from three independent experiments.
