# Supplementary material for: cAMP activates calcium signalling via phospholipase C to regulate cellulase production in the filamentous fungus Trichoderma reesei
Source: Biotechnol Biofuels. 2021 Mar 8;14:62. doi: 10.1186/s13068-021-01914-0 (PMC7941909; doi:10.1186/s13068-021-01914-0)
Supplement: Supplementary file 13 — Additional file 13: Table S5. The log2 fold changes of the putative GPCRs genes under Mn2+/DMF addition conditions. [file 13068_2021_1914_MOESM13_ESM.docx]

**Supplementary Table S5. The log_2_ fold changes of the putative GPCRs genes under Mn^2+^/DMF addition conditions.**

| **Gene ID** | **GPCR class** | **log_2_ fold change (Mn^b^ vs** **WT^a^)** | **log_2_ fold change (DMF^c^ vs** **WT^a^)** | **Regulate** |
| --- | --- | --- | --- | --- |
| Trire2_72004 | cAMP receptor-like | 1.789554995 | NO | up |
| Trire2_37525 | GPCR comprising RGS-domain | 3.799086299 | NO | up |
| Trire2_81383 | GPCR comprising RGS-domain | 3.372313788 | 3.1683528491697 | up |
| Trire2_110339  Trire2_124113  Trire2_121990 | PTH11-like  PTH11-like  PTH11-like | 5.464287319  1.198046498  1.172000898 | NO  NO  NO | up  up  up |
| Trire2_62462 | PTH11-like | 1.585953782 | NO | up |

^a^ WT, the gene expression level the wild-type strain QM6a with no addition.

^b^ Mn, the gene expression level in the wild-type strain QM6a with 10 mM Mn^2+^ addition.

^c^ DMF, the gene expression level in the wild-type strain QM6a with 1% DMF addition.

NO, no significant.
