# Supplementary material for: cAMP activates calcium signalling via phospholipase C to regulate cellulase production in the filamentous fungus Trichoderma reesei
Source: Biotechnol Biofuels. 2021 Mar 8;14:62. doi: 10.1186/s13068-021-01914-0 (PMC7941909; doi:10.1186/s13068-021-01914-0)
Supplement: Supplementary file 14 — Additional file 14: Table S6. Primers used in this study. [file 13068_2021_1914_MOESM14_ESM.docx]

| **Supplementary Table S6 Primers used in this study.**   \| Primer \| oligos Sequences (5’ to 3’) \| \| --- \| --- \|   **Construction of *Tracy1* gene deletion vector** | |
| --- | --- | --- | --- |
| acy1-D1 | ATTACGAATTCTTAATTAACTCTTTGCCCTCTGCCTGTGA |
| acy1-D2 | CATTATACGAAGTTATTCTAGACATTGCTTCCTCGTTGCCAGA |
| acy1-D3 | ACTAGTGAGCTCATTTCGGAGTTGAGAAGAGGAGGTT |
| acy1-D4 | AGTGCCAAGCTTATTTAAAGGTTCGCCAGTTTGTTGAT |
| **Verification of the *Tracy1* gene deletion mutants** | |
| acy1-CF | TTTGGGTGCGTTCGTGTGC |
| acy1-CR | ACTATTGCTGCCGTGAGGAACT |
| acy1-OF | GCGGTTCTGGACTGCGGTAA |
| acy1-OR | TGGTGCTTGCTATCGGTCTTGA |
| acy1-T1 | AACGCAAACGCAAATGCAAA |
| acy1-T2 | GCTGGCAAACGTGACACTTA |
| acy1-T3 | CAATCATGGTCGGGAGGGAT |
| acy1-T4 | AAAGACAACGTTCGCCGTAG |
| sar1-3 | GGAGGACTCGCTGGCTTCTT |
| sar1-4 | AGGATAGCAACTCGGTCGTTCT |
| **Quantitative RT-PCR analysis** | |
| Qsar1-1 | TGGATCGTCAACTGGTTCTACGA |
| Qsar1-2  Qcbh1-1  Qcbh1-2 | GCATGTGTAGCAACGTGGTCTTT  CTCCATCTCCGAGGCTCTTACC  GCAAGTGCCGCCATATCTGTTAT |
| Qegl1-1 | GCAGCCTCACCATGAACCAGTA |
| Qegl1-2 | CACCGTCAGAGTCCAGGAGATAC |
