# Supplementary material for: cAMP activates calcium signalling via phospholipase C to regulate cellulase production in the filamentous fungus Trichoderma reesei
Source: Biotechnol Biofuels. 2021 Mar 8;14:62. doi: 10.1186/s13068-021-01914-0 (PMC7941909; doi:10.1186/s13068-021-01914-0)
Supplement: Supplementary file 15 — Additional file 15: Table S7. Sequencing statistics for whole transcriptome shotgun sequencing results from this study. [file 13068_2021_1914_MOESM15_ESM.docx]

**Supplementary Table S7. Sequencing statistics for whole transcriptome shotgun sequencing results from this study.**

| Sample | Raw Reads | Clean Reads | Raw Bases(Gb) | Clean Bases(Gb) | Effective Rate (%) | Mapped Reads |
| --- | --- | --- | --- | --- | --- | --- |
| WT-1 | 46006536 | 44342540 | 6.90 | 6.65 | 96.38 | 41,623,363(93.87%) |
| WT-2 | 50019484 | 49087666 | 7.50 | 7.36 | 98.14 | 46,316,299(94.35%) |
| Mn-1 | 50211828 | 49262724 | 7.53 | 7.39 | 98.11 | 46,703,495(94.80%) |
| Mn-2 | 50042650 | 49184410 | 7.51 | 7.38 | 98.28 | 46,378,180(94.29%) |
| DMF-1 | 47644208 | 46759924 | 7.15 | 7.01 | 98.14 | 44,330,453(94.80%) |
| DMF-2 | 50345166 | 49423624 | 7.55 | 7.41 | 98.17 | 46,928,934(94.95%) |

WT: wild-type strain QM6a with no addition performed in duplicate (indicated by −1 and −2).

Mn: wild-type strain QM6a with 10 mM Mn^2+^ addition performed in duplicate (indicated by −1 and −2).

DMF: wild-type strain QM6a with 1% DMF addition performed in duplicate (indicated by −1 and −2).
